# Microglia-derived IL-1β contributes to axon development disorders and synaptic deficit through p38-MAPK signal pathway in septic neonatal rats

**DOI:** 10.1186/s12974-017-0805-x

**Published:** 2017-03-14

**Authors:** Qianpeng Han, Qiongyu Lin, Peixian Huang, Mengmeng Chen, Xin Hu, Hui Fu, Shaoru He, Fengcai Shen, Hongke Zeng, Yiyu Deng

**Affiliations:** 1 Southern Medical University, Guangzhou, 510515 People’s Republic of China; 2 Department of Critical Care and Emergency, Guangdong General Hospital, Guangdong Academy of Medical Sciences, Guangzhou, 510080 People’s Republic of China; 3 Shantou University Medical College, Shantou, Guangdong 515063 People’s Republic of China; 4 Department of Critical Care Medicine, Yueyang First People’s Hospital, Yueyang, 414000 People’s Republic of China; 5 Department of Neonatology, Guangzhou General Hospital, Guangdong Academy of Medical Sciences, Guangzhou, 510080 People’s Republic of China; 6 Department of Anatomy, Basic medical school of Wuhan University, Wuhan, Hubei 430071 People’s Republic of China

**Keywords:** Microglia, LPS, IL-1β, PWMD, Axon, Neurofilament, Synapse

## Abstract

**Background:**

Axon development plays a pivotal role in the formation of synapse, nodes of Ranvier, and myelin sheath. Interleukin-1β (IL-1β) produced by microglia may cause myelination disturbances through suppression of oligodendrocyte progenitor cell maturation in the septic neonatal rats. Here, we explored if a microglia-derived IL-1β would disturb axon development in the corpus callosum (CC) following lipopolysaccharide (LPS) administration, and if so, whether it is associated with disorder of synapse formation in the cerebral cortex and node of Ranvier.

**Methods:**

Sprague-Dawley rats (1-day old) in the septic model group were intraperitoneally administrated with lipopolysaccharide (1 mg/kg) and then sacrificed for detection of IL-1β, interleukin-1 receptor (IL-1R_1_), neurofilament-68, neurofilament-160, and neurofilament-200, proteolipid, synaptophysin, and postsynaptic density 95 (PSD95) expression by western blotting and immunofluorescence. Electron microscopy was conducted to observe alterations of axonal myelin sheath and synapses in the cortex, and proteolipid expression was assessed using in situ hybridization. The effect of IL-1β on neurofilament and synaptophysin expression in primary neuron cultures was determined by western blotting and immunofluorescence. P38-MAPK signaling pathway was investigated to determine whether it was involved in the inhibition of IL-1β on neurofilament and synaptophysin expression.

**Results:**

In 1-day old septic rats, IL-1β expression was increased in microglia coupled with upregulated expression of IL-1R_1_ on the axons. The expression of neurofilament-68, neurofilament-160, and neurofilament-200 (NFL, NFM, NFH) and proteolipid (PLP) was markedly reduced in the CC at 7, 14, and 28 days after LPS administration. Simultaneously, cortical synapses and mature oligodendrocytes were significantly reduced. By electron microscopy, some axons showed smaller diameter and thinner myelin sheath with damaged ultrastructure of node of Ranvier compared with the control rats. In the cerebral cortex of LPS-injected rats, some axo-dendritic synapses appeared abnormal looking as manifested by the presence of swollen and clumping of synaptic vesicles near the presynaptic membrane. In primary cultured neurons incubated with IL-1β, expression of NFL, NFM, and synaptophysin was significantly downregulated. Furthermore, p38-MAPK signaling pathway was implicated in disorder of axon development and synaptic deficit caused by IL-1β treatment.

**Conclusions:**

The present results suggest that microglia-derived IL-1β might suppress axon development through activation of p38-MAPK signaling pathway that would contribute to formation disorder of cortical synapses and node of Ranvier following LPS exposure.

**Electronic supplementary material:**

The online version of this article (doi:10.1186/s12974-017-0805-x) contains supplementary material, which is available to authorized users.

## Background

Neonatal sepsis may cause a systemic inflammatory response which is an important risk factor for periventricular white matter (PWM) damage (PWMD) in the developing brain [1–3]. A large number of immune effector cells are mobilized into the neonatal circulation [1–3]. Concomitantly, these immune cells release proinflammatory cytokines such as tumor necrosis factor (TNF-α) and interleukin-1β (IL-1β) which readily cross the blood-brain barrier into the brain parenchyma [4]. In the latter, the serum-derived proinflammatory cytokines can activate microglia, the resident immune cells in the central nervous system (CNS), which initiates complex inflammatory cascades including excessive release of proinflammatory cytokines, reactive oxygen species (ROS), and glutamate excitotoxicity [5, 6]. It has been reported that this may induce the injury of immature oligodendrocytes and axons resulting in hypomyelination, a hallmark feature of PWMD [7–9].

Various inflammatory mediators play different roles in the pathogenesis of PWMD [10–16]. Among them, the most widely studied mediators are the proinflammatory cytokines including TNF-α and IL-1β [17, 18]. TNF-α produced by activated microglia may elicit the apoptosis of oligodendrocytes via its TNFR_1_ which activate the signal pathway of apoptosis in the oligodendrocytes [19, 20]. There is also mounting evidence suggesting that IL-1β is a crucial contributor to various acute and chronic neurodegenerative diseases [21–23]. Unlike TNF-α, IL-1β was documented as being nontoxic to oligodendrocyte lineage cells in that it could not induce oligodendrocyte apoptosis through its receptors [24]. However, some studies have demonstrated that IL-1β can suppress oligodendrocyte proliferation at the late developmental stage of oligodendrocyte progenitor cell (OPC) [24]. Our previous studies have found that microglia-derived IL-1β could affect OPC maturation and induce hypomyelination in the PWM of septic neonatal brain [5]. In primary cultured neurons administrated with recombinant IL-1β, a significant increase in the phosphorylation of neuronal tau was accompanied by a decline in synaptophysin levels [25]. IL-1 receptor antagonist (IL-1ra) and anti-IL-1β antibody attenuated the effects of IL-1β on neuronal tau and synaptophysin [25]. Systemic inflammation activated innate immune response in the CNS and induced the release of IL-1β from activated microglia, which increased axon injury and synaptic deficit [26–29]. However, the underlying molecular mechanisms whereby IL-1β is involved in PWMD in septic neonatal rats have not been fully addressed. Here, we provide evidences that IL-1β produced by activated microglia could induce disorder of axon development and synaptic deficit in septic neonatal brain. Expression of IL-1β in microglia and its receptor 1 on developing axons was first observed by double immunofluorescence. The axon development, node of Ranvier, and myelin sheath in the PWM and synapse formation in the cerebral cortex were examined in septic rats in comparison with the controls. Furthermore, the signaling pathway via which IL-1β could suppress axon development and synapse formation was investigated. It is suggested that microglia-derived IL-1β may have a negative impact on axon development and synapse formation through activation of p38-MAPK signaling pathway after LPS administration.

## Methods

### Animals

One hundred and thirty SD rats (1-day old) provided by the Experimental Animal Center of Sun Yat-sen University were used. They were randomized into the control group and septic experimental group. The rats (*n* = 65) in the septic model group were intraperitoneally administrated with lipopolysaccharide (LPS) (1 mg/kg) derived from *Escherichia coli* 055:B5 (Sigma-Aldrich, St. Louis, MO, USA Cat. No. L2880). The rats were then housed in an animal house at room temperature for 6 h, and 2, 4, 6, 7, 14, and 28 days before being used for experiments (Table 1). The rats in the control group (*n* = 65) (Table 1) were intraperitoneally injected with equal volume of 0.01-M phosphatebuffer saline (PBS). Only male rats at 1 day of age were used for the study when the sex of rats can be determined with certainty. All animals were handled according to the protocols of Institutional Animal Care and Use Committee, Guangdong Province, China.

**Table 1:** Number of rats killed at various time points after the LPS exposure (in brackets) and their age-matched controls for various methods (outside the brackets)

Control(LPS)	Immunofluorescence	Western blotting	In situ hybrization	Electron microscopy
6 h	3(3)	5(5)		
2 days	3(3)	5(5)		
4 days	3(3)	5(5)		
6 days	3(3)	5(5)		
7 days	3(3)	5(5)		
14 days	3(3)	5(5)	3(3)	
28 days	3(3)	5(5)	3(3)	3(3)

### Primary cultures of cortical neurons

Primary cortical neuron culture was performed using neonatal SD rats (1-day old), as described previously [30] with some modifications. The cerebral cortices were dissected from the neonatal brain, minced into small tissue pieces of size 1 mm^3^ excluding the hippocampus and meninges, trypsinized for 15 min with 0.125% trypsin (Gibco) at 37 °C, and then neutralized with fetal bovine serum (FBS) (Life Technology). Cells were dissociated by passage through a pasteur pipette. The suspension containing neural cells was centrifuged at 1100 rpm for 5 min; thereafter, the cells were resuspended in Dulbecco’s modified eagle medium (DMEM) containing 10% fetal bovine serum (Life Technology) and plated in 6-well plates (Corning) for various experiments, or plated in 96-well plates (Corning) specifically for the CCK-8 test. All the plates were pre-coated with poly-l-lysine (Sigma). At 6 h after plating, DMEM/10% FBS was replaced with neurobasal medium containing 2% B27 and 1% glutamine; half of the medium was replaced with neurobasal containing 2% B27 without glutamine 3 days later. For immunocytochemistry, primary cultured neurons were detached from 75-cm^2^ flask, then plated at a density of 2.5 × 10^5^/well in a 24-multiwell culture dish. For western blotting, primary cultured neurons were plated at 1 × 10^6^ cells per flask; thereafter, they received different treatments according to experimental protocols on the following day. The purity of cortical neurons was examined through immunocytochemical staining using MAP-2 (a marker of neurons) and 4′6-diamidino-2-phenylindole (DAPI). The purity of primary neuron cultures in this study was above 95%.

### Treatment of primary cultured neurons

To determine the IL-1β concentration, the CCK-8 assay (cell counting kit 8) was performed following the manufacturer’s instructions (DOJINDO). Primary cultured neurons (3000 cells/well in 100-ul neurobasal medium) were incubated with different concentrations (0, 5, 10, 20, 40, 80, 100, and 200 ng/ml) of IL-1β for 24 h in 96-well plates (Corning) at 37 °C. Ten-microliter CCK-8 solution was then added to each well, and the plates were incubated at 37 °C for 6 h. The optical density (OD) in each well was measured at 450 nm using an enzyme-linked immunosorbent assay (ELISA) reader (BioTek, Winooski, VT, USA) according to the manufacturer’s instructions. The neuronal cell activity remained relatively changed when neurons were treated with IL-1β at a dose less than 40 ng/mL (Additional file 1: Figure S1).

Primary neuron cultures were divided into three groups as follows:

- Group I
  To examine the effects of IL-1β on expression of NFL, NFM, and synaptophysin in primary cultured neurons by western blotting analysis and immunocytochemical staining, the cells were cultured in neurobasal medium containing 2% B27 and 1% glutamine in a humidified atmosphere of 95% air and 5% CO_2_ for 24 h. The neurons were plated in 6-well plates at the density of 2 × 10^6^/well for western blotting analysis and in 6-well plates at the density of 1.2 × 10^6^/well for immunofluorescence staining. The neurons in group I were randomized into four groups including the control group (0.01 M PBS), IL-1β (40 ng/mL) group, IL-1β (40 ng/mL) + IL-1Ra (40 ng/mL) group, and IL-1Ra (40 ng/mL) group.
- Group II
  The primary cultured cortical neurons in group II were used to investigate the effects of IL-1β on the phosphorylation of p38-MAPK pathway. For this, the primary neurons were administrated with 40-ng/ml IL-1β for 0.5, 1, 2, 4, and 6 h before harvest.
- Group III
  To determine whether p38-MAPK signaling pathway is implicated in the effects of IL-1β on expression of NFL, NFM, and synaptophysin in primary cultured cortical neurons, the cells (2 × 10^6^/well) were cultured in 6-well plates in a humidified atmosphere of 95% air and 5% CO_2_ at 37 °C. Subsequently, the cells were randomized into four subgroups including the control group (0.01 M PBS), IL-1β (40 ng/ml) group, IL-1β (40 ng/ml) + SB203580 (10 μmol/L) group, and SB203580 (10 μmol) group. After incubation with above-mentioned reagents for 24 h, the cells were harvested.

### Western blot

A protein extraction kit (Pierce Biotechnology Inc, IL, USA) was used to extract proteins from the cortex or the corpus callosum or primary cultured cortical neurons following the standard protocol. Protein concentrations were measured according to the bicinchonininc acid (BCA) method [31]. Standard western blot protocols were followed as described in a previous study by us [19]. The primary antibodies used (Table 2) were as follows: IL-1β, interleukin-1 receptor (IL-1R_1_), NFL, NFM, NFH, synaptophysin, postsynaptic density 95 (PSD95), PLP, p38, Phos-p38, and β-actin. Following three rinses in tris-buffered saline Tween (TBST), the membranes were hybridized with the horseradish peroxidase (HRP)-conjugated secondary antibodies (1:1000, Cell Signaling Technology; Cat. No 7074 (anti-rabbit IgG) or 7076 (anti-mouse IgG)) for 2 h at 4 °C. The enhanced chemiluminescence detection system (Pierce Biotechnology Inc, Rockford, IL, USA) was used to develop the immunoblots on the membranes. Subsequently, the immunoblots were stripped using the stripping buffer (Pierce Biotechnology Inc.; Cat. No. 0021059). Following this, the membranes were incubated with total kinase or β-actin. The signal intensity of the respective protein bands was calculated with FluorChem 8900 software, version 4.0.1 (Alpha Innotech Corporation, San Leandro, CA, USA), and the fold change relative to control was measured.

**Table 2:** Antibodies used in experiments

Antibody	Host	Company	Cat.No.	Application	Concentration
Phos-p38	Rabbit	Cell Sigaling Technology, Danvers, MA, USA	4511S	Cell (WB)	1:1000
p38	Rabbit	Cell Sigaling Technology, Danvers, MA, USA	8690S	Cell (WB)	1:1000
β-actin	Mouse	Cell Sigaling Technology, Danvers, MA, USA	3700S	Tissue/cell (WB)	1:1000
IL-1β	Rabbit	Chemicon International, Temecula, CA, USA	AB1832P	Tissue (WB/IF)	1:1000
IL-1R_1_	Rabbit	Santa Cruz Biotechnology, Santa Cruz, CA, USA	SC689	Tissue (WB/IF)	1:100
NFH	Mouse	Sigma-Aldrich, Saint Louis, MO, USA	N0142	Tissue/cell (WB)	1:1000
NFM	Mouse	Sigma-Aldrich, Saint Louis, MO, USA	N2787	Tissue/cell (WB/IF)	1:1000
NFL	Mouse	Sigma-Aldrich, Saint Louis, MO, USA	N5139	Tissue/cell (WB/IF)	1:1000
MAP-2	Mouse	Abcam, Cambridge, MA, USA	ab11267	Cell (IF)	1:500
Synaptophysin	Mouse	Abcam, Cambridge, MA, USA	ab8049	Tissue (IF)	1:200
Synaptophysin	Rabbit	Abcam, Cambridge, MA, USA	ab14692	Tissue/cell (WB)	1:200
PSD-95	Rabbit	Abcam, Cambridge, MA, USA	ab18258	Tissue (WB/IF)	1:500
PLP	Rabbit	Abcam, Cambridge, MA, USA	ab28486	Tissue (WB/IF)	1:500
Alexa Fluor555	Goat	Invitrogen Life Technologies Corporation	A-21422	Tissue/cell (IF)	1:200
Alexa Fluor555	donkey	Invitrogen Life Technologies Corporation	A-31572	Tissue/cell (IF)	1:200
Alexa Fluor488	donkey	Invitrogen Life Technologies Corporation	A-21202	Tissue/cell (IF)	1:200
Lectin		Sigma-Aldrich, St. Louis, MO	L2886	Tissue/cell (IF)	1:100

### Immunofluorescence

Coronal frozen brain sections of 10-μm thickness were incubated with 0.3% hydrogen peroxide in methanol to deactivate endogenous peroxidase for 20 min. After washing three times with PBS, the sections were blocked with a mixed solution composed of 5% BSA and 0.3% Triton X-100 in PBS for 30 min at room temperature. Subsequently, the brain sections from rats at different time points (*n* = 3 at each time point) after PBS or LPS injection were randomized into five groups. The sections in group I from rats at 7, 14, and 28 days after PBS or LPS injection were incubated with antibody against NFL (Table 2). The sections in group II from the control and septic rats at 14 and 28 days were incubated with PLP antibody (Table 2). The sections in group III from the control and septic rats at 14 and 28 days were incubated with antibodies against PSD95 (Table 2) and synaptophysin (Table 2). The sections in group IV from rats at 6 h and 2, 4, and 6 days after PBS and LPS injection were incubated with IL-1β antibody (Table 2) and Lectin (Table 2). The brain sections in group V from the control and septic rats at 2 and 4 days were incubated with antibody against IL-1R_1_ (Table 2) and NFL (Table 2). On the next day, after washing three times with PBS, the sections were incubated with secondary antibodies: Alexa Fluor555 goat anti-mouse IgG (H + L) (Table 2) for NFL in group I, Alexa Fluor555 donkey anti-rabbit IgG (H + L) (Table 2) for PLP in group II, Alexa Fluor555 donkey anti-rabbit IgG (H + L) (Table 2) and Alexa Fluor488 donkey anti-mouse IgG (H + L) (Table 2) for PSD95/synaptophysin in group III and for IL-1R_1_/NFL in group V, and Alexa Fluor555 donkey anti-rabbit IgG (H + L) (Table 2) for IL-1β in group IV for 1 h. After three rinses in PBS, the sections in group IV were incubated with Lectin (Table 2) for 1 h. Incubation of sections for all groups was carried out at room temperature. Finally, all sections were counterstained with DAPI (Sigma-Aldrich, St. Louis, MO, USA, Cat. No. D9542) and then observed using a fluorescence microscope (Olympus System Microscope Model BX53, Olympus Company Pte, Tokyo, Japan).

For primary cultured cortical neurons, the cells were treated with protein IL-1β, IL-1β + IL-1Ra, IL-1Ra, and the equal volume of PBS for 1 day. After three rinses in PBS, the cells were fixed in 4% paraformaldehyde for 30 min and then blocked in 1% BSA for 1 h. After this, the cells were incubated with NFM antibody (Table 2) overnight at 4 °C. On the next day, after three rinses in PBS (10 min each time), the cells were incubated with Alexa Fluor488 donkey anti-mouse IgG (H + L) (Table 2) for 1 h. Following three washes in PBS, the cells were incubated with DAPI for 5 min and observed under a fluorescence microscope (Olympus System Microscope Model BX53, Olympus Company Pte, Tokyo, Japan).

### Electron microscopy

LPS-injected rats (*n* = 3 at 28 days) and littermate controls (*n* = 3 at 28 days) were transcardially perfused with a mixed aldehyde fixative composed of 2% paraformaldehyde and 3% glutaraldehyde. Coronal sections of the brain at about 1-mm thick were prepared. Blocks of the corpus callosum (CC) and cerebral cortex were trimmed from the brain slices. These blocks were cut into vibratome sections of 80–100-μm thickness by a vibratome (Model 3000™, The Vibratome™ Company, St Louis, MO, USA). The vibratome sections were then washed overnight in 0.1-M phosphate buffer, postfixed for 2 h in 1% osmium tetroxide, dehydrated, and embedded in Araldite mixture. Ultrathin sections, doubly stained with uranyl acetate and lead citrate, were observed under a Philips CM 120 electron microscope (FEI™ Company, Hillsboro, OR, USA). Four different areas of the CC or cerebral cortex from each of the brain were scrutinized and photographed at three different magnifications. Image J software (SummaSketch III Summagraphics, Seattle, WA) was used to measure the diameter of each axon magnified at 6800 times by a blind researcher.

### In situ hybridization

We performed in situ hybridization on 10-μm-thick coronal frozen brain sections as previously described [32, 33]. Briefly, brain sections were incubated with proteinase K (S3004, Dako, Carpinteria, CA, USA) for 10 min and then rinsed in distilled water in 96% ethanol and in isopropanol for 5 min each. Following this, the brain sections were incubated with 125 μL of hybridization mixtures composed of 15 μL of distilled water, 25 μL of 20× saline-sodium citrate (SSC) buffer, 62.5 μL of 50% formamide, 12.5 μL of 50% dextran sulfate, 2.5 μL of Denhardt’s solution (D2532, Sigma-Aldrich, Saint Louis, MO,USA), 6.25 μL of herring sperm DNA (D7290, Sigma-Aldrich), and 1.25 μL of 3′-digoxigenin-conjugated probe (presented from prof fu hui’ lab). The probe, 5′-CAAGGGAAGGGAGGAAGAGACAG-3′, in final concentration of 100 ng/mL, detects a segment of the 5.8S ribosomal RNA of PLP. The sections were incubated at 95 °C for 6 min, immediately chilled in ice, and then incubated at 40 °C for 14–16 h in a humidified chamber. After this, the sections were washed in 2× SSC, 1× SSC, and 0.1× SSC buffer for 5 min each, followed by incubation with the anti-digoxigenin antibody conjugated to alkaline phosphatase, diluted in tris-buffered saline (TBS) (1:200, 11093274910, Roche Diagnostics, Indianapolis, IN, USA) for 1 h, and then washed in TBS. Visualization was achieved using NBT/BCIP (nitro blue tetrazolium/5-bromo-4-chloro-3-indolyl-phosphate) (11681451001, Roche Diagnostics) for 1 h in the dark. The reaction was stopped with TE buffer (pH 8.0) for 10 min and then washed in distilled water. The sections were counterstained with Mayer’s hematoxylin and mounted in aqueous medium (Faramount, S3025, Dako). PLP-positive cells were examined under a microscope (Olympus System Microscope Model BX53, Olympus Company Pte, Tokyo, Japan). Four nonoverlapping regions of the corpus callosum from each animal were photographed at two different magnifications. PLP-positive cells were enumerated under ×10 magnifications.

### Statistical analysis

The present data were analyzed by the SPSS 20.0 statistical software (IBM, Armonk, New York, USA). The results were presented as mean ± SD. Statistical significance was examined by Student’s *t* test. The statistical significance of the results was considered at *P* < 0.05.

## Results

### Neurofilament protein expression in the CC

**Fig. 1:**
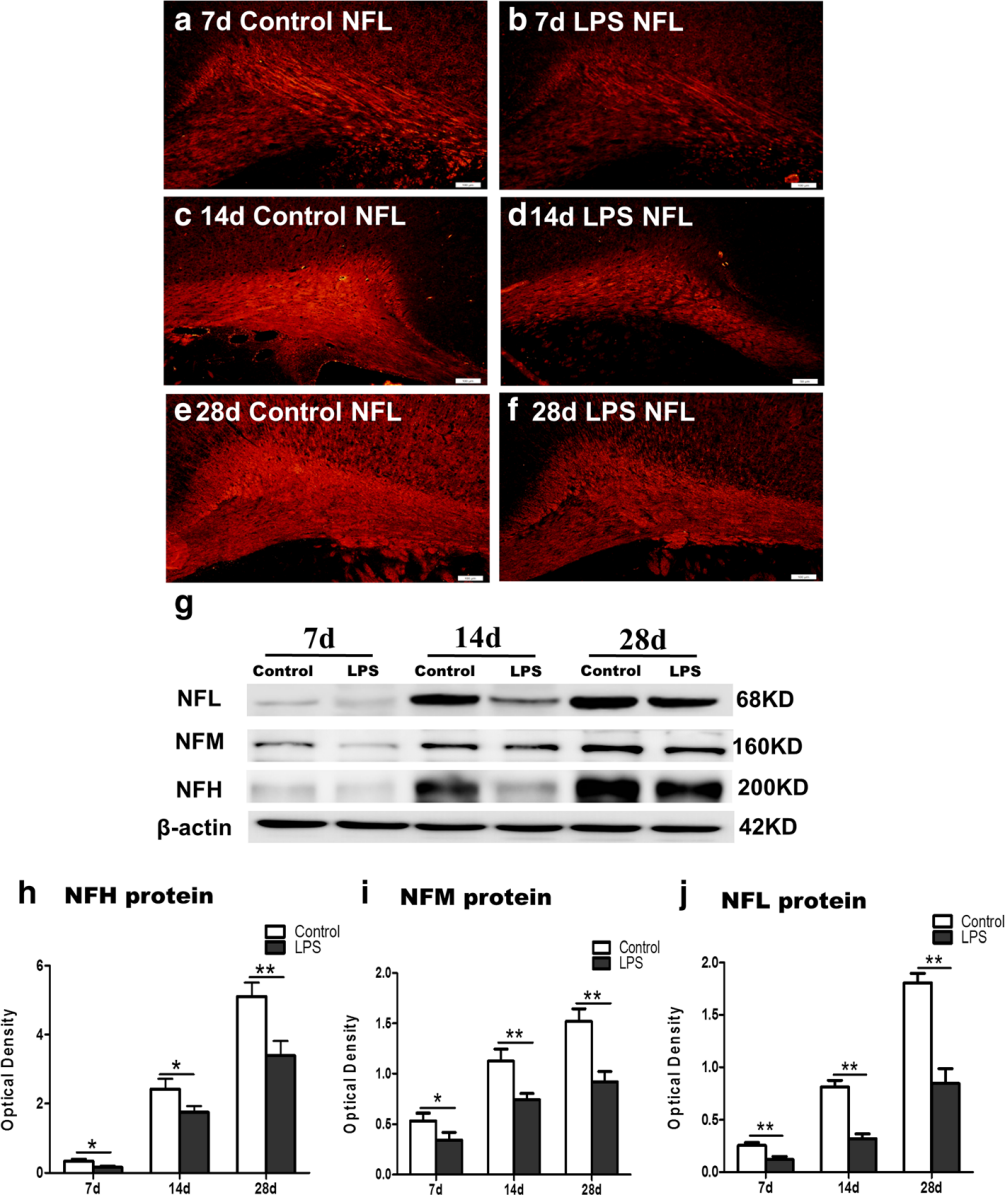
LPS inhibits axon development. **a**–**j** show NFL, NFM, and NFH protein expression in the PWM of postnatal rats at 7, 14, and 28 days after LPS injection and their corresponding controls. Immunofluorescene shows NFL expression in PWM of postnatal rats at 7, 14, and 28 days after LPS injection (**b**, **d**, and **f**) and their corresponding controls (**a**, **c**, and **e**). **g** The immunoactive bands of NFL (68 kDa), NFM (160 kDa), NFH (200 kDa), and β-actin (42 kDa) by western blot analysis. **h**–**j** Bar graphs depicting significant decrease in the optical density of NFL, NFM, and NFH expression, respectively, following LPS challenge when compared with matched control. *P < 0.05, **P < 0.01. Scale bars: **a**–**f** 100 μm

NFL, NFM, and NFH protein expression was markedly decreased in the CC at 7, 14, and 28 days after LPS administration when compared with the matching controls (Fig. 1a–j). The optical density of immunoreactive bands of NFL, NFM, and NFH protein expression was significantly reduced at the same time points in LPS-injected rats in comparison with the matching controls (Fig. 1g–j). Immunostaining showed that NFL expression was noticeably reduced in the CC at 7, 14, and 28 days after LPS administration (Fig. 1a–f).

### Number of neurons in the cerebral cortex

To ascertain if there was a significant loss of neurons in the whole cortex in the frontal lobe after LPS treatment, the sections from the control and septic rats at 7, 14, and 28 days were incubated with antibodies against NeuN and Caspase-3, an apoptotic marker. After LPS treatment, the number of mature and apoptotic neurons at 7, 14, and 28 days in the cerebral cortex (Additional file 2: Figure S2D–F, J–L, and P–R) was comparable to that in the matching controls (Additional file 2: Figure S2A–C, G–I, and M–O). To confirm this, Nissl’s staining was carried out in sections from the control and septic rats at 7, 14, and 28 days. The results showed that the number of neurons was not significantly changed in the cerebral cortex in the frontal lobe at 7, 14, and 28 days after LPS injection (Additional file 3: Figure S3B, D, and F) in comparison with the corresponding control (Additional file 3: Figure S3A, C, and E).

### Ultrastructural study

**Fig. 2:**
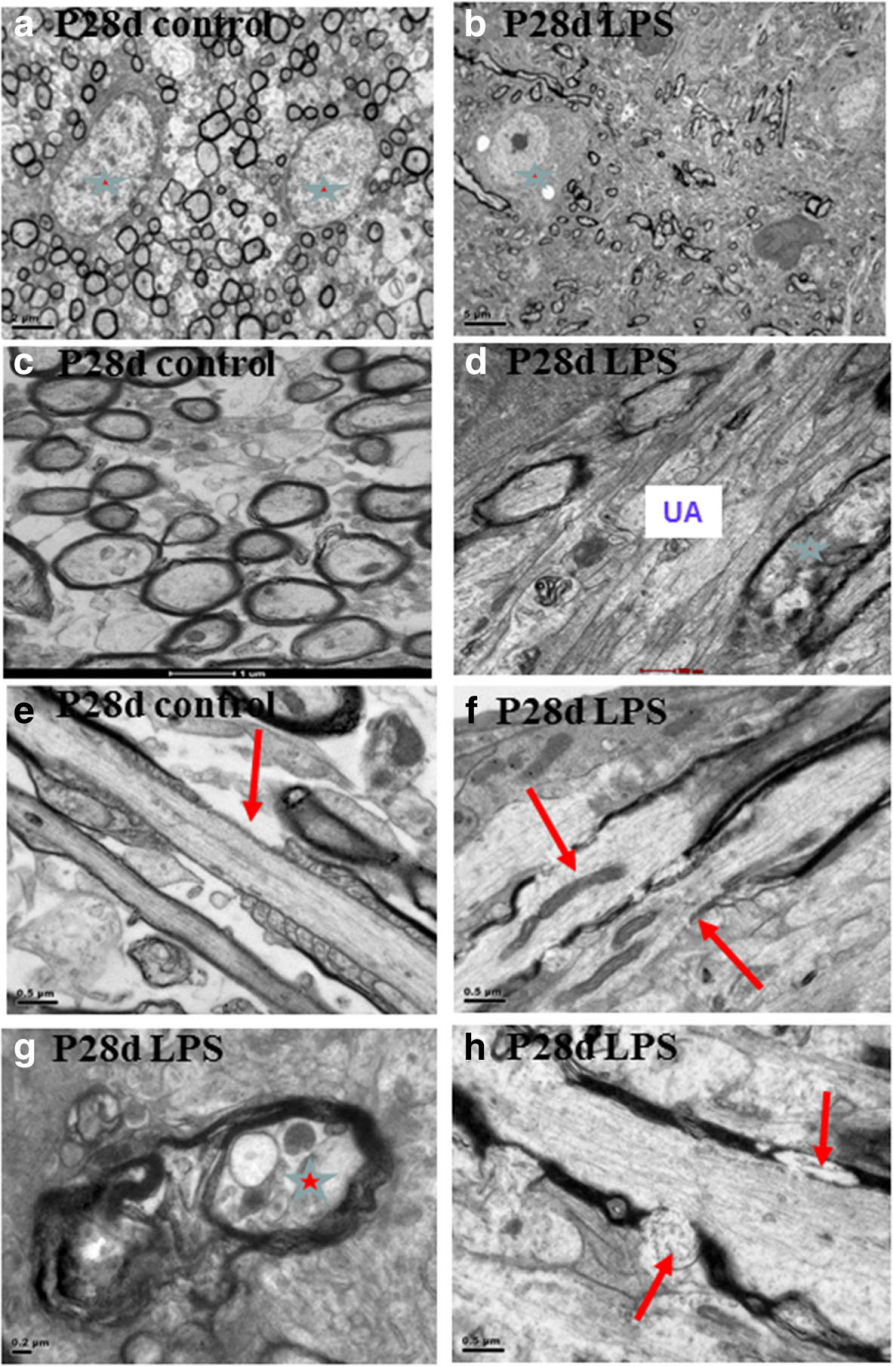
Electron micrographs show thinner axons and aberrant Ranver node in PWM of 28-day rats. Electron microscopic images of PWM in cross-sections are shown at different magnifications. **a** Myelinated axons are regularly packed in the control with intact two oligodendrocytes (*asterisks*) under low magnification. **b** Note drastic reduction in myelinated axons at 28 days after LPS injection. Normal Oligodendrocyte (*asterisk*)) appears less. **c** Myelinated axons in the control. Note the thick myelin sheath under high magnification. **d** Note drastic reduction in myelinated axons at 28 days after LPS injection and some are distorted (*asterisk*). Many unmyelinated axons (UA) are seen. **e** Normal node of Ranvier (*arrow*) in the control. **f** Disrupted node of Ranvier (*arrows*) at 28 days after LPS injection. **g** Disrupted axon (*asterisk*) and myelin sheath at 28 days after LPS injection. **h** Disrupted myelin sheath (*arrows*) at 28 days after LPS injection. Scale bars: **a** 2 μm; **b** 5 μm; **c** 1 μm; **d** 500 nm; **e**–**f** 0.5 μm; **g** 0.2 μm; **h** 0.5 μm

By electron microscopy, at 28 days after LPS injection, the packing density of myelinated axons was decreased significantly in the CC (Fig. 2b, d) when compared with the corresponding controls (Fig. 2a, c) under low and high magnification. Indeed, in LPS-injected rats, many axons appeared unmyelinated (Fig. 2b, d). Additionally, in LPS-injected rats, the diameter of axons was noticeably smaller at 28 days in comparison with that of the matching controls. The average axonal diameter in 28 days control rats was 2.84 ± 0.12 μm. It was obviously reduced to 1.91 ± 0.10 μm in rats with LPS treatment, indicating thinner axons in the septic rat brain. Ultrastructural study also showed damaged myelin sheath (Fig. 2g, h) and Ranvier node at 28 days after LPS injection (Fig. 2f) (arrow) when compared with the intact myelin sheath and Ranvier node (Fig. 2e) (arrow) in the corresponding control.

**Fig. 3:**
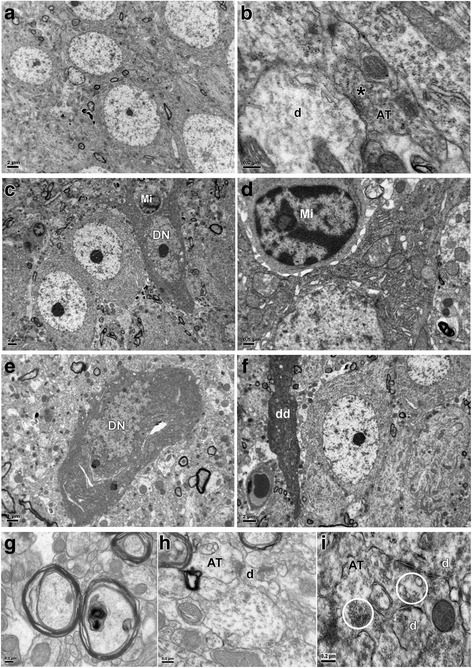
Electron micrographs. Few normal neurons in the cerebral cortex in a control rat (**a**). They show a round or oval nucleus with fine and discrete chromatin clumps. Profiles of myelinated axons are seen in the neuropil. At a higher magnification (**b**), axon terminals (AT) makes synaptic contact with dendrites (d) are common. Note the presence of a granular synaptic vesicles (*asterisk*) in the terminal; synaptic membrane thickening is evident. In rats given LPS injection at 28 days (**c**), some neurons show enhanced electron density in both the soma and dendrites (DN). An activated microglia (Mi) is seen closely associated with the soma of a “darkened neuron” (**c**, **d**). In an enlarged view (**d**), the “darkened neuron” showed dilated profiles of rough endoplasmic reticulum and mitochondria with disrupted cristae. The nucleoplasm of the “darkened neuron” also shows increased density (**e**). Profiles of “darkened dendrites” (dd) often appear to course through the neuropil (**f**). In the neuropil are present myelinated axons with disrupted myelin and contain dense inclusions (**g**). Axo-dendritic synapses in which some synaptic vesicles (*circled*) are swollen, clumped, and aggregated near the presynaptic membrane (**h**, **i**). AT, axon terminal; d, dendrite, DN, darkened neuron; Mi, microglia. Scale bars are indicated in the respective images

The neurons in the cerebral cortex above the CC appeared relatively normal in the P28d control rats by electron microscopy (Fig. 3a). The neurons were characterized by a round to oval nucleus containing mainly euchromatin. Profiles of myelinated axons and dendrites were present in the neuropil. Axo-dendritic synapses were readily identified (Fig. 3b), while axo-somatic synapses were less common. In LPS-injected rats, sacrificed at 28 days, some neurons showed enhanced electron density affecting both the soma and dendrites (Fig. 3c–f). On closer examination, the “darkened neurons” showed dilated cisternae of rough endoplasmic reticulum and mitochondria. In the neuropil of LPS-injected rats, profiles of myelinated axons containing dense inclusions and with disrupted myelin sheath were observed (Fig. 3g). Some axo-dendritic synapses appeared abnormal looking as manifested by the presence of swollen and clumping of synaptic vesicles near the presynaptic membrane (Fig. 3h, i).

### PLP protein expression in the CC

**Fig. 4:**
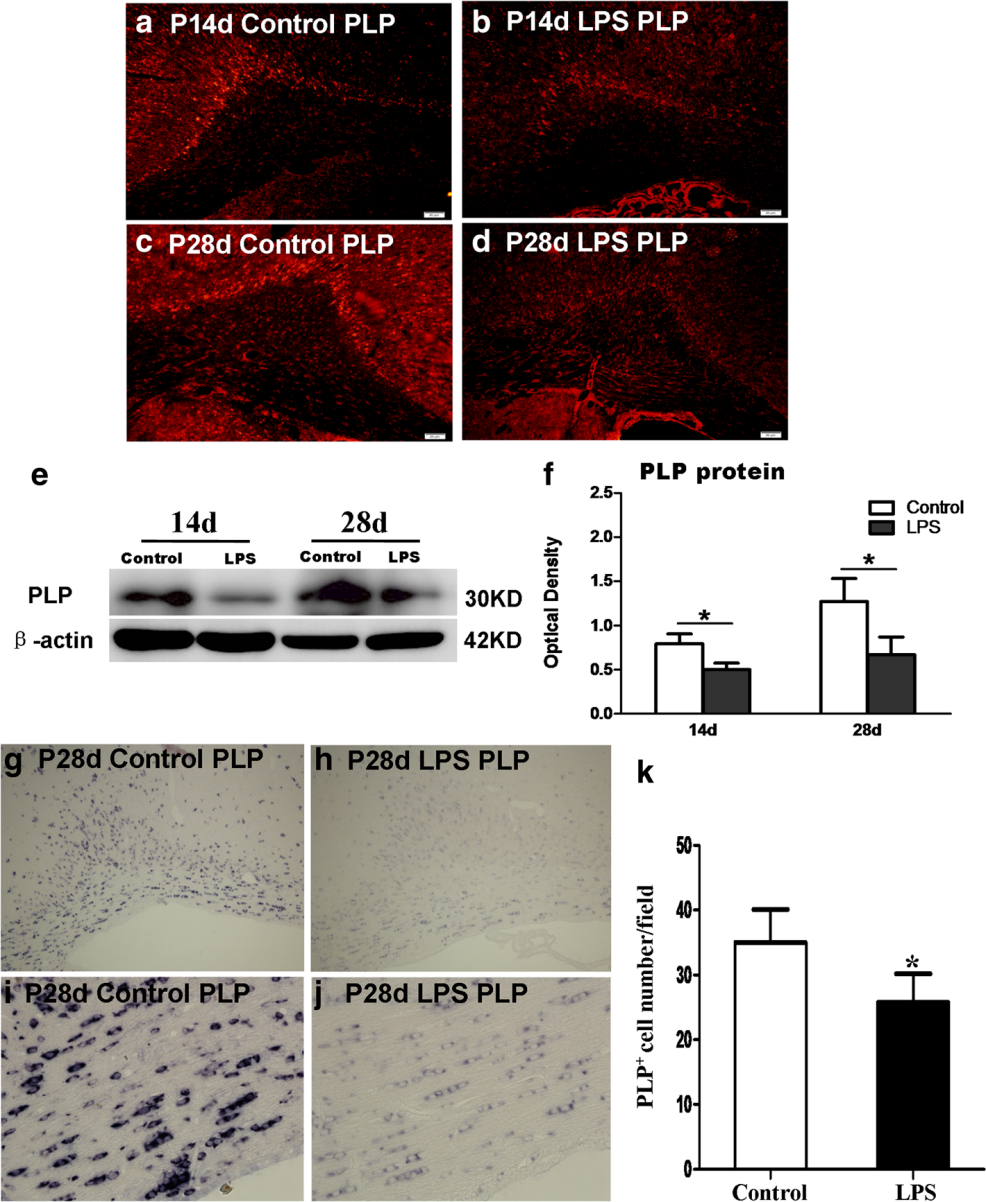
LPS inhibits the maturation of oligodendrocytes in the PWM at 14 and 28d. **a**–**f** show PLP protein expression in the PWM at 14 and 28d after LPS injection and the matching controls. Immunofluorescene images showing the expression of PLP in the PWM at 14 and 28d of the LPS exposure (**b**, **d**) and corresponding control rats (**a**, **c**). **e** PLP (30 kDa) and β-actin (42 kDa) immunoreactive bands. Bar graph (**f**) shows significant reduction in the optical density of PLP following LPS exposure in comparison with the corresponding controls (*P < 0.01). In situ hybridization in (**g**–**k**) showing the number of PLP^+^ oligodendrocytes in the PWM at 28 days after LPS exposure and the matching control. Panel (**g**, **h**) the number of PLP^+^ oligodendrocytes in the PWM under low microscopy (×10). Panel (**i**, **j**) the number of PLP^+^ oligodendrocytes in the PWM under high microscopy (×40). Bar graph (**k**) showing the number of PLP^+^ oligodendrocytes in the PWM. Note that the number of PLP^+^ oligodendrocytes in the PWM at 28 days after LPS exposure decreased significantly when compared with the corresponding controls (^*^ *P* <0.01). Scale bars: **a**–**d** 40 μm

PLP is a specific marker for mature myelin sheath in CNS that is produced by oligodendrocytes. Immunostaining showed that PLP immunoreactivity was downregulated at 14 and 28 days in the CC of LPS-injected rats in comparison with their corresponding control littermates (Fig. 4a–d). The immumoreactive band of PLP protein levels that appeared at approximately 30 kDa (Fig. 4e) was evidently reduced in the optical density at 14 and 28 days in LPS-injected rats in comparison with the age-matched controls (Fig. 4e, f). The marked reduction of PLP expression was confirmed by immunostaining and western blot analysis. In situ hybridization was performed with antisense riboprobes to PLP in the CC of 28-day control (Fig. 4g, i) and LPS-injected rats (Fig. 4h, j). PLP expression was obviously decreased in the CC at 28 days following LPS injection (Fig. 4h, j). The number of PLP-positive oligodendrocytes was concomitantly reduced (Fig. 4h, k).

### Synaptophysin and PSD95 protein expression in the cortex

**Fig. 5:**
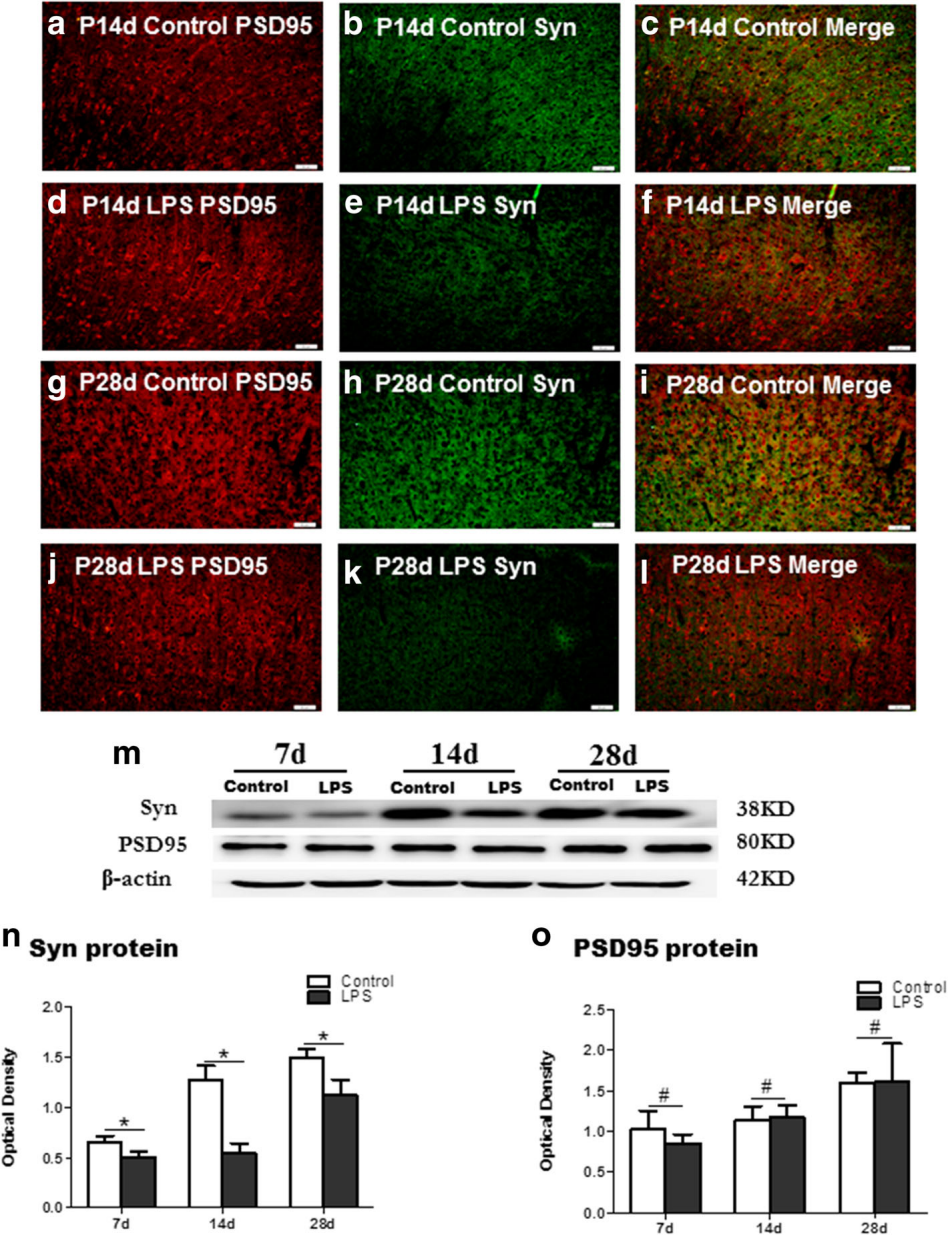
LPS elicits excitatory synaptic deficits in the cerebral cortex. Synapses were identified by the punctate co-labeling of the excitatory synaptic proteins, synaptophysin (**b**, **e**, **h**, and **k**), and PSD-95 (**a**, **d**, **g**,and **j**) in the cerebral cortex. LPS exposure reduced the number of synaptophysin/PSD-95 puncta in the cerebral cortex at 14 and 28 days (**f**, **l**) when compared with the corresponding control (**c**, **i**). Panel (**m**) shows synaptophysin (38 kDa), PSD-95 (80 kDa), and β-actin (42 kDa) immunoreactive bands, respectively. Bar graph (**n**) shows significant decrease in the optical density of synaptophysin in the cerebral cortex at 7, 14, and 28 days following LPS injection in comparison with their matching controls. Bar graph (**o**) shows the optical density of PSD-95 in the cerebral cortex at 7, 14, and 28 days following LPS injection was not significantly different in comparison with their matching controls. *P < 0.05, #*P* > 0.05. Scale bars: **a**–**l** 50 μm

Immunofluorescence staining showed that synaptophysin immunoreactivity was reduced in the cortex at 14 and 28 days following LPS injection in comparison with the age-matched controls (Fig. 5b, e, h, and k). PSD95 protein expression at 14 and 28 days was comparable between LPS-injected rats and age-matched control rats (Fig. 5a, d, g, and j). Western blotting showed a significant decrease in synaptophysin immunoreactivity at 7, 14, and 28 days in the cortex of LPS-injected rats (Fig. 5m, n). However, PSD95 protein expression remained relatively unchanged at 7, 14, and 28 days in the cortex in the septic rats when compared with the controls (Fig. 5m, o).

### IL-1β and IL-1R_1_ protein expression in the CC

**Fig. 6:**
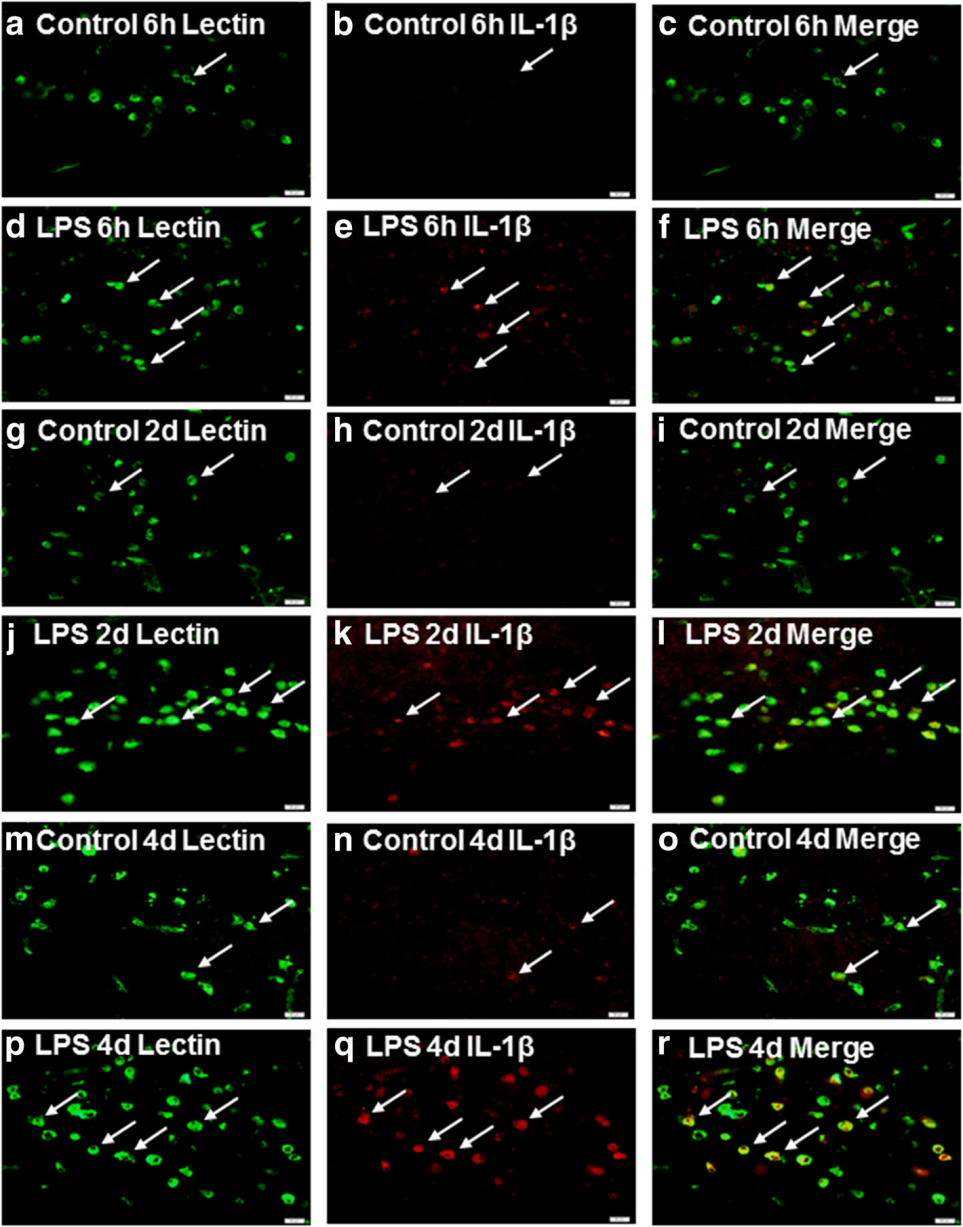
IL-1β is expressed in the amoeboid microglial cells (AMCs) in the corpus callosum *in vivo*. Lectin-labeled (**a**, **d**, **g**, **j**, **m**, and **p**, *green*) and IL-1β (**b**, **e**, **h**, **k**, **n**, and **q**, *red*) immunoreactive AMCs are distributed in the corpus callosum at 6 h and 2 and 4 days after the LPS injection and their matching controls. Lectin labeling clearly overlaps IL-1β immunofluorescence (**c**, **f**, **i**, **l**, **o,** and **r**). Note that IL-1β expression in AMCs is noticeably enhanced after the LPS exposure. Scale bars: **a**–**r** 20 μm

**Fig. 7:**
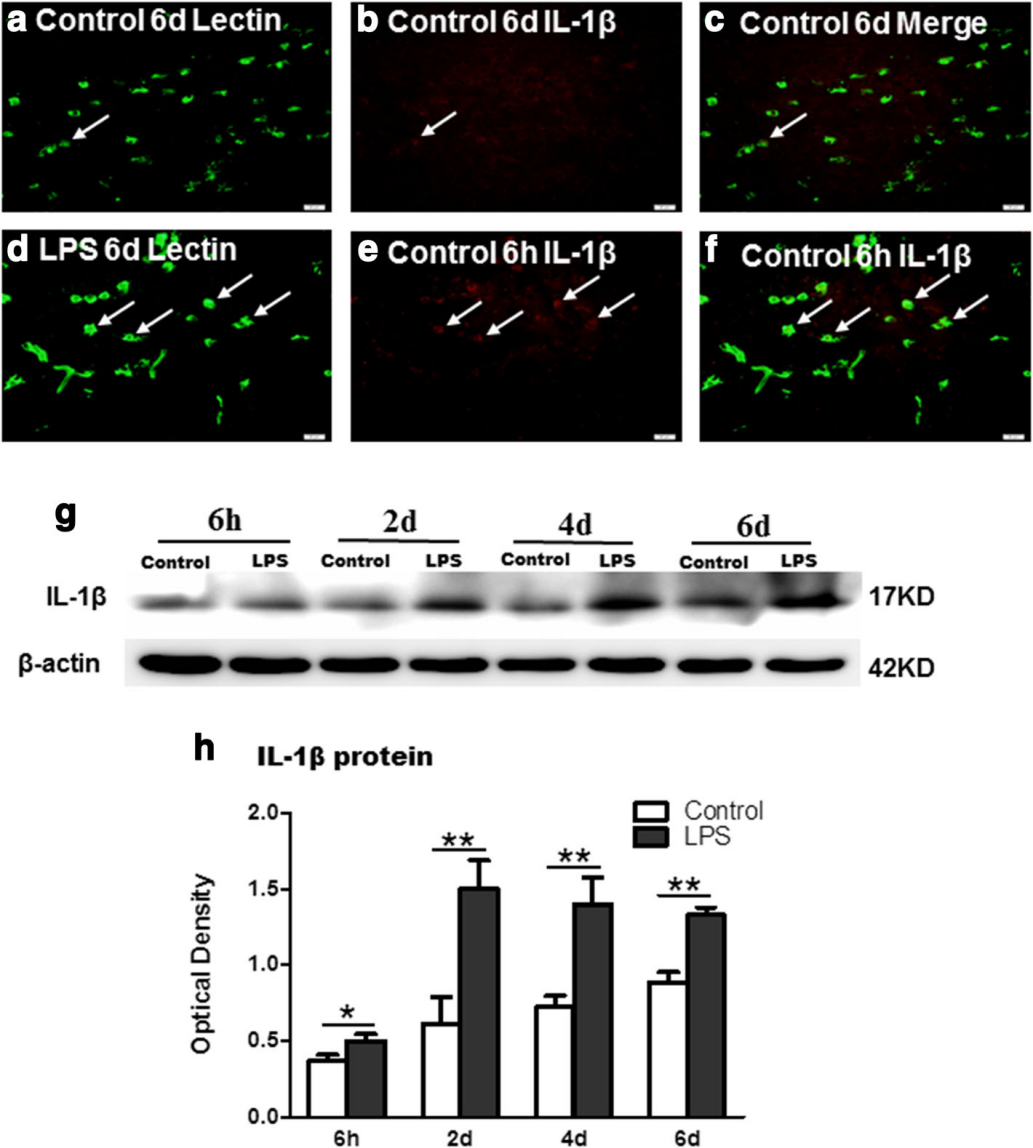
IL-1β protein expression in the corpus callosum of postnatal rats at 2 h and 2, 4, and 6 days after LPS exposure and their corresponding controls. Panel (**a**–**f**) show that IL-1β immunoreactive cells (**b**, **e**, *red*) clearly overlap lectin-labeled AMCs (**a**, **d**, *green*) in (**c** and **f**) in the corpus callosum of neonatal rats at 6 days after the LPS exposure and their corresponding controls. Panel (**g**) shows IL-1β (17 kDa) and β-actin (42 kDa) immunoreactive bands. Bar graph (**h**) shows significant increase in optical density of IL-1β following LPS injection in comparison with their matching controls. *P < 0.05, **P < 0.01. Scale bars: **a**–**f** 20 μm

**Fig. 8:**
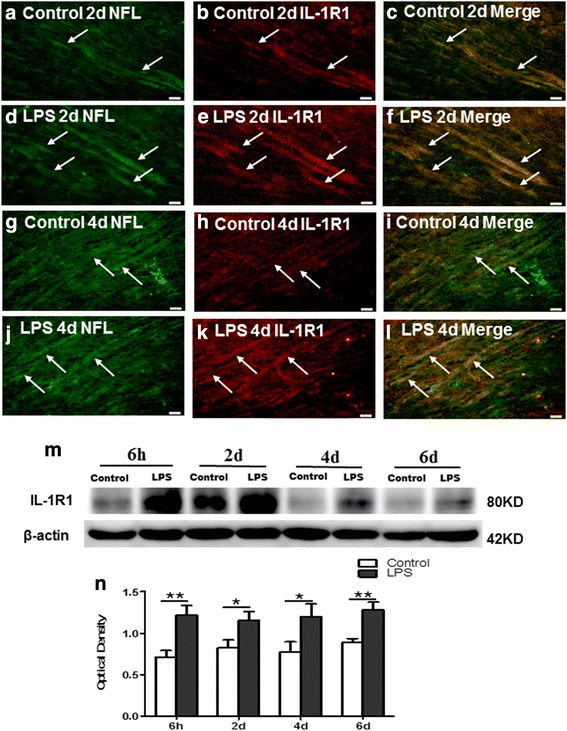
IL-1R_1_ protein expression in the PWM of neonatal rats at 6 h and 2, 4, and 6 days after the LPS injection and their matching controls. Immunofluorescene images show the distribution of IL-1R_1_ (**b**, **e**, **h**, and **k,** *red*) in NFL immunoreactive axons (**a**, **d**, **g**, and **j** *green*) in the PWM at 2 and 4 days after the LPS exposure and the matching control. Co-localized expression of NFL with IL-1R_1_ is depicted in panels (**c**, **f**, **i,** and **l**). Panel (**m**) shows IL-1R_1_ (80 kDa) and β-actin (42 kDa) immunoreactive bands. Bar graph (**n**) shows significant changes in the optical density of IL-1R_1_ following LPS exposure when compared with the corresponding control. Note the expression of IL-1R_1_ is significantly increased after LPS exposure. *P < 0.05, **P < 0.01. Scale bars: **a**–**l** 20 μm

Double immunofluorescence showed that IL-1β expression was specifically localized in lectin-labeled amoeboid microglial cells (AMCs) in the CC (Figs. 6a–r and 7a–f). At 6 h and 2, 4, and 6 days following LPS intraperitoneal injection, IL-1β immunoreactivity was enhanced in large numbers of AMCs (Figs. 6d–f, j–l, and p–r; 7d–f) when compared with the corresponding controls (Figs. 6a–c, g–i, and m–o; 7a–c). The optical density of IL-1β immumoreactive band was markedly upregulated at 6 h and 2, 4, and 6 days after LPS administration in comparison with the controls (Fig. 6g, h). Double immunofluorescence showed that IL-1R_1_ immunoreactivity was detected on the NFL-labeled axons (Fig. 8a–l). IL-1R_1_ protein expression was significantly increased along the closely packed axons at 2 and 4 days following LPS exposure in the CC (Fig. 8a–c, d–f, g–i, and j–l). The optical density of IL-1R_1_ immumoreactive band that appeared at approximately 80 kDa (Fig. 8m) was significantly upregulated at 6 h and 2, 4, and 6 days following LPS exposure (Fig. 8m, n).

### NFL, NFM, NFH, and synaptophysin immunoreactivity in primary cortical neuron cultures following administration with IL-1β

**Fig. 9:**
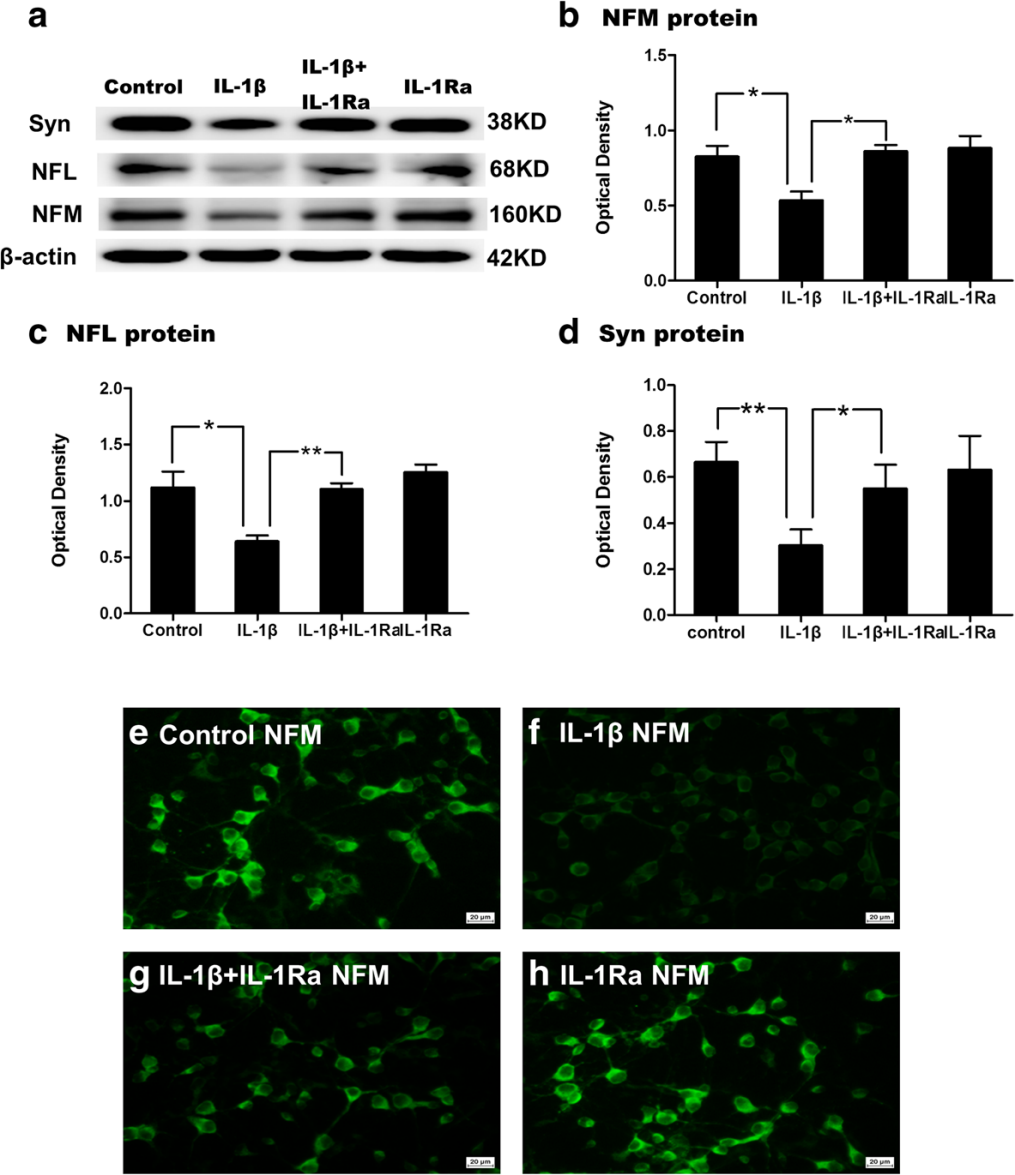
IL-1β impedes the protein expression of NFM, NFL and synaptophysin in vitro. Panel (**a**) shows NFM (160 kDa), NFL (68 kDa) and synaptophysin (38 kDa) immunoreactive bands. Bar graphs in (**b**, **c**, and **d**) show changes in the optical density of NFM, NFL and synaptophysin, respectively, following IL-1β administration. The protein expression of NFM, NFL and synaptophysin is significantly reduced in neuron culture at 24 h after IL-1β administration when compared with the corresponding controls. IL-1R antagonist reversed the reduction. (*P < 0.05, **P < 0.01). Immunofluorescene images show the expression of NFM in neuron culture at 24 h after IL-1β (**f**), IL-1β + IL-1Ra (**g**), or IL-1Ra (**h**) administration when compared with the control (**e**). Scale bars: **e**–**h** 100 μm

To determine the expression levels of various neuronal proteins after IL-1β treatment, assay using CCK-8 (cell counting kit 8) was performed. When the dosage of IL-1β exceeded 40 ng/mL, expression of neuronal proteins was drastically decreased (Additional file 1: Figure S1). Expression of various proteins was affected when neurons were treated with IL-1β less than 40 ng/mL. Therefore, IL-1β at 40 ng/mL was used in this study for determination of its effects on neural proteins. To explore the effects of IL-1β on development of neurofilament and formation of synapse in primary cortical neurons in vitro, the immunoreactivity of NFL, NFM, NFH, and synaptophysin was evaluated in primary cortical neurons in mixed culture medium for 24 h administered with IL-1β, IL-1β + IL-1Ra, IL-1Ra, and equal volume of PBS as control, respectively. Immunofluorescence staining showed that immunoreactivity for NFM was decreased at 24 h after IL-1β administration (Fig. 9f) in comparison with the controls (Fig. 9e). Administration of IL-1R antagonist obviously reverted the reduction of NFM protein expression induced by IL-1β treatment (Fig. 9g). In addition, administration of IL-1R antagonist alone did not alter the protein expression of NFM (Fig. 9h). The optical density of NFL, NFM, and synaptophysin immunoreactive band was markedly reduced at 24 h following IL-1β administration (Fig. 9a–d). Administration of IL-1R antagonist reversed the decreased expression of NFL, NFM, and synaptophysin induced by IL-1β (Fig. 9a–d).

### Effect of IL-1β on p38-MAPK signaling pathway in primary cortical neurons

**Fig. 10:**
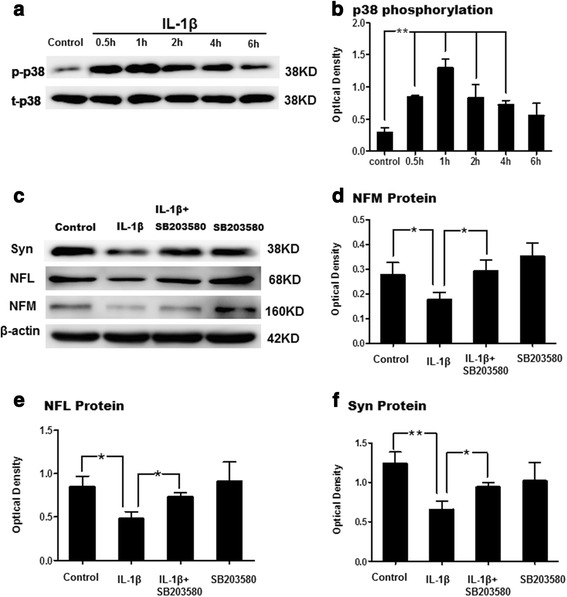
IL-1β inhibits NFL, NFM and synaptophysin protein expression via activation of p38-MAPK signaling pathway in primary cultured cortical neurons. Panel (**a**) shows p38 phosphorylation and total p38 (38KDa) immunoreactive bands. Panels (**b**) is a bar graph depicting significant changes in the optical density of p38 phosphorylation at different time points following IL-1β administration (**P<0.01). Panels (**c**) shows NFM (160 kDa), NFL (68 kDa), and synaptophysin (38 kDa) immunoreactive bands, indicating that SB203580 (a p38 inhibitor) may reverse the reduction of NFM, NFL and synaptophysin expression after IL-1β administration. Bar graphs in (**d**, **e**, and **f**) show significant increase in expression of NFM, NFL and synaptophysin, respectively, by SB203580. (*P <0.05, **P<0.01)

Phosphorylated p38 level was examined in primary cortical neurons incubated with IL-1β (40 ng/ml) for 0, 0.5, 1, 2, 4, and 6 h by western blot analysis. The expression level was markedly upregulated at 0.5 h, peaking at 1 h but gradually declined to basal levels at 6 h after 1 L-1β treatment (Fig. 10a–b). Furthermore, IL-1β administration did not affect the total p38 level at all time points (Fig. 10a–b).

### IL-1β inhibited the protein expression of NFL, NFM, and synaptophysin through promoting the phosphorylation of p38-MAPK pathway in primary cortical neurons

To verify that IL-1β could suppress NFL, NFM, and synaptophysin expression in primary cortical neurons by activating the p38-MAPK pathways, NFL, NFM, and synaptophysin protein levels were detected by western blotting in primary cortical neurons treated with IL-1β + SB203580, a selective inhibitor of p38. Incubation of primary cortical neurons with SB203580 30 min prior to IL-1β administration for 24 h reversed the inhibition of NFL, NFM, and synaptophysin protein expression induced by IL-1β (Fig. 10c–f).

## Discussion

There are five principal subunit proteins of neuron-specific intermediate filaments (IFs) including the light, medium, and heavy molecular mass neurofilament (NF) triplet proteins (NFL, NFM, and NFH, respectively), α-internexin, and peripherin [34–36]. Mature filaments are made up of different combinations of these five subunits [37]. NFL, NFM, and NFH are the main cytoskeletal elements in mature neurons, although NFH expression is usually delayed relative to NFL and NFM [38, 39]. Their main role is to increase the axonal caliber of myelinated axons and consequently axonal conduction velocity [38, 39]. NFM and NFL are required for axonal radial growth [40–42]. Loss of NFM and NFL results in small caliber axons [40–42]. The axons are wrapped by many processes of oligodendrocytes to form myelin sheath in the CNS [43]. The axolemma is relatively uncovered at regularly spaced nodes of Ranvier [44]. These structures allow rapid and efficient saltatory propagation of action potentials along Ranvier nodes, which enhance information transmission on axons [45]. The formation of synapses occurs between axons and dendrites of different neurons in the CNS [46]. Synaptic proteins including synaptophysin and post-synaptic density-95 (PSD-95) are involved in synaptic plasticity [46]. Synaptophysin is localized in presynaptic vesicle membranes, which play important roles in docking, fusion, endocytosis, and membrane trafficking [47]. PSD-95 is a post-synaptic protein, which is associated with regulating the number and size of dendritic spines and developing glutamatergic synapses [48]. Changes in these synaptic proteins have been used to evaluate synaptic deficit [49]. The present results have shown that NFL, NFM, and NFH protein expression level was significantly reduced in the CC at different times after LPS injection. By electron microscopy, a lesser number and smaller diameter of axons were observed. A striking structural feature or alteration in the LPS-injected rats was the occurrence of some “darkened neurons” which were not present in the cerebral cortex of control rats. Interestingly, some of the “darkened neurons” were seen in juxtaposition to normal looking neurons which have also been found in ischemic monkey brain [50, 51]. Therefore, it can be confidently argued that they were not fixation artifacts rather they represent degenerating neurons which have also been reported in traumatic brain injuries in the primates [52]. In light of this, it is suggested that LPS injection had caused damage or structural alterations to some neurons in the developing cerebral cortex as well as their associated synapses. As a result, it is possible that the neuronal proteins including those that constitute the synapses in the cortex would be altered. The close association of activated microglia with the “darkened neuron” may not be fortuitous. LPS is known to stimulate microglia in the brain and their release of proinflammatory cytokines such as IL-1β that might have induced the neuronal changes affecting the soma, axons, dendrites, and synapses as demonstrated in this study. It is suggested that these are associated with decreased level of synaptophysin, Ranvier node structural damage, and reduction of mature oligodendrocytes. In other words, axon development in the CC was inhibited in the septic brain that ultimately would lead to presynaptic deficit, axonal hypomyelination, along with Ranvier node structural damage.

It is well documented that complex crosstalks occur between developing axons and oligodendrocyte progenitor cell processes during myelin sheath formation [53]. Normal axon development is indispensable for oligodendrocyte proliferation, maturation, and subsequent myelination [53]. The present results have shown that PLP protein level was drastically downregulated in the CC in septic brain coupled with reduction in the number of PLP-positive oligodendrocytes. It stands to reason therefore that inhibition of axon development can cause disorder of oligodendrocyte maturation and hypomyelination in the CC in LPS-injected rats.

We show here that microglia, especially those closely associated with the callosal axons in the CC, were activated and generated excess amounts of IL-1β after LPS injection. Microglia activation may be elicited by serum-derived proinflammatory mediators which have gained access to the brain tissue by passing through the disrupted blood-brain barrier [15]. Interestingly, microglial activation was sustained up to nearly a week, suggesting that it is a persistent and intense inflammatory response in the CC in septic rats. Furthermore, at a late stage of the inflammatory response, the reactive astrocytes would be an additional cellular source for IL-1β [54]. Besides IL-1β, activated microglia release other proinflammatory mediators in adverse conditions including TNF-α, inducible nitric oxide synthase (iNOS), and NO; all these have been reported to cause the loss of oligodendrocyte and myelination deficit through the corresponding signaling pathways [19, 28, 55]. The exhibition of IL-1R_1_ expression on NFL immunoreactive axons was augmented and remained to be so for about a week after LPS challenge. It has been documented that IL-1β exerts direct inhibitory effect on axonal growth of developing superior cervical ganglion sympathetic neurons via activating NF-κB signaling pathway [56]. In agreement with this, IL-1β exerted an inhibitory effect on the outgrowth of axons from cultured dorsal root ganglion cells in vitro [57]. In light of this, it was surmised that IL-1β derived from the microglia might suppress the development of axons via IL-1R_1_ in the PWM of septic neonatal rats. We show here reduction of NFM, NFL in the CC, and synaptophysin expression in the cerebral cortex in vivo. However, the number of neurons was not decreased significantly in the cortex, albeit the identification by electron microscopy of some “darkened neurons” indicative of neuronal degeneration or death. Additionally, IL-1β administration in vitro was found to decrease expression of NFM, NFL, and synaptophysin in primary neurons. Remarkably, IL-1 receptor antagonist neutralized the inhibitory effect of IL-1β on expression of NFM, NFL, and synaptophysin. Taken together, these results suggest that IL-1β treatment could inhibit axonal development and synapse formation in primary culture neurons. More importantly, the in vitro results corroborated with in vivo findings.

**Fig. 11:**
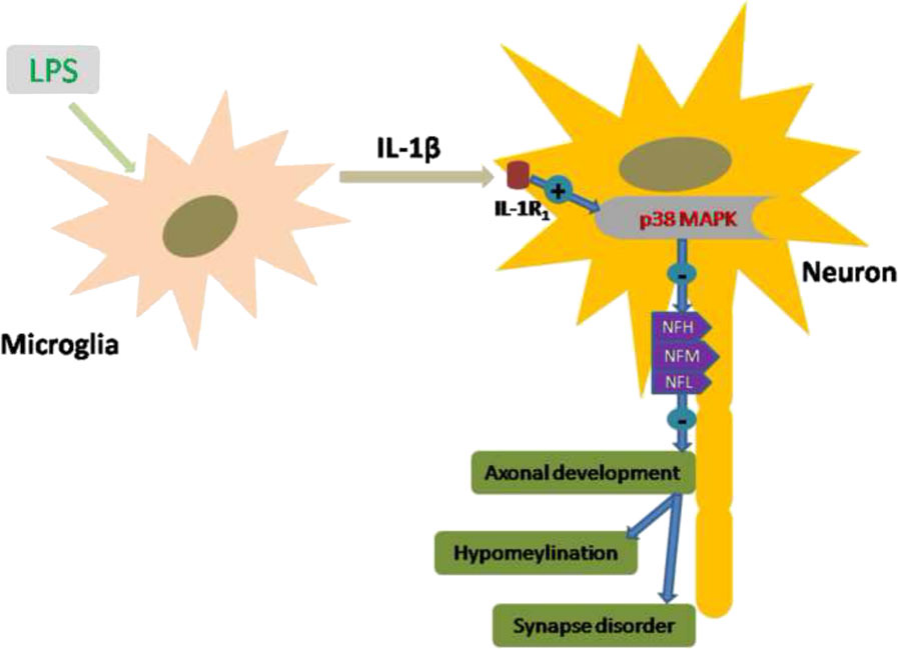
TOCI. An illustration demonstrates the cellular and molecular events associated with PWMD in the septic developing brain. Microglia are activated after LPS intraperitoneal injection and release proinflammatory cytokine IL-1β, which inhibits the generation of NFL, NFM, and NFH in the axon through its IL-1R_1_ via p38-MAPK signaling pathway. This suppresses axon development and contributes to axonal hypomyelination in septic neonatal brain. Moreover, the synaptogenesis of neurons involved in this process is ultimately affected

Previous studies have reported that increased p38-MAPK activity may be responsible for the loss of synaptophysin following LPS administration [25, 58, 59]. In addition, inhibition of p38-MAPK activity can revert the loss of synaptophysin induced by IL-1β in rat primary cortical neuronal cultures and in an animal model of Alzheimer’s disease [25, 58, 59]. We have shown that the phosphorylated p38 levels were markedly increased at 0.5 h and gradually declined to basal levels at 6 h in the primary cultured neurons treated with IL-1β protein. Administration of SB203580, a selective inhibitor of p38, upregulated NFM, NFL, and synaptophysin protein levels that were suppressed by IL-1β incubation. On the basis of these findings, it is concluded that IL-1β might inhibit the expression of NFM, NFL, and synaptophysin through IL-1R_1_-p38-MAPK signaling pathway in primary neurons; hence, inhibition of p38-MAPK signaling pathway may help ameliorate axon injury and synaptic deficit in the septic neonatal brain (Fig. 11).

## Conclusions

This study has shown reduction of axonal neurofilament protein expression coupled with disorder of axonal myelin sheath formation and synaptic deficit in the PWM and cerebral cortex, respectively, of septic developing brain. Concomitantly, AMCs associated with the axons were activated and produced a large amount of IL-1β. The possible crosstalk between AMCs and axons through IL-1β and its receptor 1 localized on axons would perturb the development of axons, which would contribute to disorder of myelin sheath formation and synaptic deficit. In vitro, IL-1β inhibited the expression of NFL, NFM, NFH, and synaptophysin in primary neurons via p38-MAPK signaling pathway. Therefore, inhibition of the biochemical and/or molecular processes mentioned above may represent one potential therapeutic strategy in mitigating PWMD induced by LPS in the developing brain.

## Additional files

Additional file 1: Figure S1. The CCK-8 assay (cell counting kit 8) was performed to determine the IL-1β concentration. The viability of neuronal cells was significantly reduced when neurons were treated with IL-1β at a dose exceeding 40 ng/mL. (TIF 820 kb)
Additional file 2: Figure S2. Apoptosis of neurons in the cortex. The incidence of apoptotic neurons co-labeled by caspase-3 (*red*) and NeuN (*green*) did not change markedly at 7 days (A–F), 14 days (G–L), and 28 days (M–R) in the cerebral cortex after LPS injection (D–F, J–L, P–R) when compared with controls (A–C, G–I, M–O). Scale bars: A–R 20μm. (TIF 6590 kb)
Additional file 3: Figure S3. Nissle staining shows the number of neurons in the cortex of postnatal rats at 7, 14, and 28 days after LPS injection (B, D, F) and their corresponding controls (A, C, E). (TIF 7048 kb)

## Abbreviations

AMCs: Amoeboid microglial cells
BCA: Bicinchonininc acid
BSA: Bovine serum albumin
CC: Corpus callosum
CCK-8: Cell counting kit 8
CNS: Central nervous system
DAPI: 4′6-diamidino-2-phenylindole
DMEM: Dulbecco’s modified eagle medium
ELISA: Enzyme-linked immunosorbent assay
FBS: Fetal bovine serum
HRP: Horseradish peroxidase
IL-1R_1_: Interleukin-1 receptor
IL-1ra: IL-1 receptor antagonist
IL-1β: Interleukin-1β
LPS: Lipopolysaccharide
MAP-2: Microtubule-associated protein-2
NBT/BCIP: Nitro blue tetrazolium/5-bromo-4-chloro-3-indolyl-phosphate
NFH: Neurofilament-200
NFL: Neurofilament-68
NFM: Neurofilament-160
OD: Opacity density
OPC: Oligodendrocyte progenitor cell
p38-MAPK: p38-mitogen-activated protein kinase
PBS: Phosphate-buffer saline
PLP: Proteolipid
PSD95: Postsynaptic density 95
PWM: Periventricular white matter
PWMD: Periventricular white matter damage
ROS: Reactive oxygen species
SSC: Saline-sodium citrate
TBS: Tris-buffered saline
TBST: Tris-buffered saline Tween-20
TNFR_1_: Tumor necrosis factor receptor
TNF-α: Tumor necrosis factor-α

## References

[1] Favrais G, van de Looij Y, Fleiss B, Ramanantsoa N, Bonnin P, Stoltenburg-Didinger G, Lacaud A, Saliba E, Dammann O, Gallego J, Sizonenko S, Hagberg H, Lelièvre V, Gressens P (2011). Systemic inflammation disrupts the developmental program of white matter. Ann Neurol.

[2] Alshaikh B, Yusuf K, Sauve R (2013). Neurodevelopmental outcomes of very low birth weight infants with neonatal sepsis: systematic review and meta-analysis. J Perinatol.

[3] Kuypers E, Ophelders D, Jellema RK, Kunzmann S, Gavilanes AW, Kramer BW (2012). White matter injury following fetal inflammatory response syndrome induced by chorioamnionitis and fetal sepsis: lessons from experimental ovine models. Early Hum Dev.

[4] Basu S, Agarwal P, Anupurba S, Shukla R, Kumar A (2015). Elevated plasma and cerebrospinal fluid interleukin-1 beta and tumor necrosis factor-alpha concentration and combined outcome of death or abnormal neuroimaging in preterm neonates with early-onset clinical sepsis. J Perinatol.

[5] Xie D, Shen F, He S, Chen M, Han Q, Fang M, Zeng H, Chen C, Deng Y (2016). IL-1β induces hypomyelination in the periventricular white matter through inhibition of oligodendrocyte progenitor cell maturation via FYN/MEK/ERK signaling pathway in septic neonatal rats. Glia.

[6] Manning SM, Talos DM, Zhou C, Selip DB, Park HK, Park CJ, Volpe JJ, Jensen FE (2008). NMDA receptor blockade with memantine attenuates white matter injury in a rat model of periventricular leukomalacia. J Neurosci.

[7] Khwaja O, Volpe JJ (2008). Pathogenesis of cerebral white matter injury of prematurity. Arch Dis Child Fetal Neonatal Ed.

[8] Billiards SS, Haynes RL, Folkerth RD, Borenstein NS, Trachtenberg FL, Rowitch DH, Ligon KL, Volpe JJ, Kinney HC (2008). Myelin abnormalities without oligodendrocyte loss in periventricular leukomalacia. Brain Pathol.

[9] Haynes RL, Billiards SS, Borenstein NS, Volpe JJ, Kinney HC (2008). Diffuse axonal injury in periventricular leukomalacia as determined by apoptotic marker fractin. Pediatr Res.

[10] Kaur C, Rathnasamy G, Ling EA (2013). Roles of activated microglia in hypoxia induced neuroinflammation in the developing brain and the retina. J Neuroimmune Pharmacol.

[11] Deng YY, Lu J, Ling EA, Kaur C (2011). Role of microglia in the process of inflammation in the hypoxic developing brain. Front Biosci (Schol Ed).

[12] Kaur C, Ling EA (2009). Periventricular white matter damage in the hypoxic neonatal brain: role of microglial cells. Prog Neurobiol.

[13] Back SA, Rivkees SA (2004). Emerging concepts in periventricular white matter injury. Semin Perinatol.

[14] Rezaie P, Dean A (2002). Periventricular leukomalacia, inflammation and white matter lesions within the developing nervous system. Neuropathology.

[15] Saliba E, Marret S (2001). Cerebral white matter damage in the preterm infant: pathophysiology and risk factors. Semin Neonatol.

[16] Deng YY, Lu J, Ling EA, Kaur C (2009). Monocyte chemoattractant protein-1 (MCP-1) produced via NF-kappaB signaling pathway mediates migration of amoeboid microglia in the periventricular white matter in hypoxic neonatal rats. Glia.

[17] Rathnasamy G, Ling EA, Kaur C (2011). Iron and iron regulatory proteins in amoeboid microglial cells are linked to oligodendrocyte death in hypoxic neonatal rat periventricular white matter through production of proinflammatory cytokines and reactive oxygen/nitrogen species. J Neurosci.

[18] Rousset CI, Chalon S, Cantagrel S, Bodard S, Andres C, Gressens P, Saliba E (2006). Maternal exposure to LPS induces hypomyelination in the internal capsule and programmed cell death in the deep gray matter in newborn rats. Pediatr Res.

[19] Deng Y, Lu J, Sivakumar V, Ling EA, Kaur C (2008). Amoeboid microglia in the periventricular white matter induce oligodendrocyte damage through expression of proinflammatory cytokines via MAP kinase signaling pathway in hypoxic neonatal rats. Brain Pathol.

[20] Wang Y, Yin P, Huang S, Wang J, Sun R (2013). Ethyl pyruvate protects against lipopolysaccharide-induced white matter injury in the developing rat brain. Int J Dev Neurosci.

[21] Wu MD, Montgomery SL, Rivera-Escalera F, Olschowka JA, O’Banion MK (2013). Sustained IL-1β expression impairs adult hippocampal neurogenesis independent of IL-1 signaling in nestin + neural precursor cells. Brain Behav Immun.

[22] Ghosh S, Wu MD, Shaftel SS, Kyrkanides S, LaFerla FM, Olschowka JA, O’Banion MK (2013). Sustained interleukin-1β overexpression exacerbates tau pathology despite reduced amyloid burden in an Alzheimer’s mouse model. J Neurosci.

[23] Rothwell N (2003). Interleukin-1 and neuronal injury: mechanisms, modification, and therapeutic potential. Brain Behav Immun.

[24] Vela JM, Molina-Holgado E, Arévalo-Martín A, Almazán G, Guaza C (2002). Interleukin-1 regulates proliferation and differentiation of oligodendrocyte progenitor cells. Mol Cell Neurosci.

[25] Li Y, Liu L, Barger SW, Griffin WS (2003). Interleukin-1 mediates pathological effects of microglia on tau phosphorylation and on synaptophysin synthesis in cortical neurons through a p38-MAPK pathway. J Neurosci.

[26] Moreno B, Jukes JP, Vergara-Irigaray N, Errea O, Villoslada P, Perry VH, Newman TA (2011). Systemic inflammation induces axon injury during brain inflammation. Ann Neurol.

[27] Di Penta A, Moreno B, Reix S, Fernandez-Diez B, Villanueva M, Errea O, Escala N, Vandenbroeck K, Comella JX, Villoslada P (2013). Oxidative stress and proinflammatory cytokines contribute to demyelination and axonal damage in a cerebellar culture model of neuroinflammation. PLoS One.

[28] Deng Y, Xie D, Fang M, Zhu G, Chen C, Zeng H, Lu J, Charanjit K (2014). Astrocyte-derived proinflammatory cytokines induce hypomyelination in the periventricular white matter in the hypoxic neonatal brain. PLoS One.

[29] Dammann O, Hagberg H, Leviton A (2001). Is periventricular leukomalacia an axonopathy as well as an oligopathy?. Pediatr Res.

[30] Li Y, Wang J, Sheng JG, Liu L, Barger SW, Jones RA, Van Eldik LJ, Mrak RE, Griffin WS (1998). S100 beta increases levels of beta-amyloid precursor protein and its encoding mRNA in rat neuronal cultures. J Neurochem.

[31] Brown RE, Jarvis KL, Hyland KJ (1989). Protein measurement using bicinchoninic acid: elimination of interfering substances. Anal Biochem.

[32] Dugas JC, Cuellar TL, Scholze A, Ason B, Ibrahim A, Emery B, Zamanian JL, Foo LC, McManus MT, Barres BA (2010). Dicer1 and miR-219 are required for normal oligodendrocyte differentiation and myelination. Neuron.

[33] Bialas AR, Stevens B (2013). TGF-β signaling regulates neuronal C1q expression and developmental synaptic refinement. Nat Neurosci.

[34] Laser-Azogui A, Kornreich M, Malka-Gibor E, Beck R (2015). Neurofilament assembly and function during neuronal development. Curr Opin Cell Biol.

[35] Lowery J, Kuczmarski ER, Herrmann H, Goldman RD (2015). Intermediate filaments play a pivotal role in regulating cell architecture and function. J Biol Chem.

[36] Shea TB, Lee S (2013). The discontinuous nature of neurofilament transport accommodates both establishment and repair of the axonal neurofilament array. Cytoskeleton (Hoboken).

[37] Fliegner KH, Kaplan MP, Wood TL, Pintar JE, Liem RK (1994). Expression of the gene for the neuronal intermediate filament protein alpha-internexin coincides with the onset of neuronal differentiation in the developing rat nervous system. J Comp Neurol.

[38] Mahammad S, Murthy SN, Didonna A, Grin B, Israeli E, Perrot R, Bomont P, Julien JP, Kuczmarski E, Opal P, Goldman RD (2013). Giant axonal neuropathy-associated gigaxonin mutations impair intermediate filament protein degradation. J Clin Invest.

[39] Shen H, Barry DM, Garcia ML (2010). Distal to proximal development of peripheral nerves requires the expression of neurofilament heavy. Neuroscience.

[40] Elder GA, Friedrich VL, Kang C, Bosco P, Gourov A, Tu PH, Zhang B, Lee VM, Lazzarini RA (1998). Requirement of heavy neurofilament subunit in the development of axons with large calibers. J Cell Biol.

[41] Zhu Q, Couillard-Després S, Julien JP (1997). Delayed maturation of regenerating myelinated axons in mice lacking neurofilaments. Exp Neurol.

[42] Garcia ML, Lobsiger CS, Shah SB, Deerinck TJ, Crum J, Young D, Ward CM, Crawford TO, Gotow T, Uchiyama Y, Ellisman MH, Calcutt NA, Cleveland DW (2003). NF-M is an essential target for the myelin-directed “outside-in” signaling cascade that mediates radial axonal growth. J Cell Biol.

[43] Ishii A, Furusho M, Dupree JL, Bansal R (2014). Role of ERK1/2 MAPK signaling in the maintenance of myelin and axonal integrity in the adult CNS. J Neurosci.

[44] Amor V, Feinberg K, Eshed-Eisenbach Y, Vainshtein A, Frechter S, Grumet M, Rosenbluth J, Peles E (2014). Long-term maintenance of Na + channels at nodes of Ranvier depends on glial contact mediated by gliomedin and NrCAM. J Neurosci.

[45] Rosenbluth J, Mierzwa A, Shroff S (2013). Molecular architecture of myelinated nerve fibers: leaky paranodal junctions and paranodal dysmyelination. Neuroscientist.

[46] Kim HJ, Thayer SA (2009). Lithium increases synapse formation between hippocampal neurons by depleting phosphoinositides. Mol Pharmacol.

[47] Evans GJ, Cousin MA (2005). Tyrosine phosphorylation of synaptophysin in synaptic vesicle recycling. Biochem Soc Trans.

[48] El-Husseini AE, Schnell E, Chetkovich DM, Nicoll RA, Bredt DS (2000). PSD-95 involvement in maturation of excitatory synapses. Science.

[49] Harigaya Y, Shoji M, Shirao T, Hirai S (1996). Disappearance of actin-binding protein, drebrin, from hippocampal synapses in Alzheimer’s disease. J Neurosci Res.

[50] Little JR, Kerr FW, Sundt TM (1974). Synaptic alterations in developing cortical infarction:an experimental investigation in monkeys. Stroke.

[51] Wang XY, Wong WC, Ling EA (1995). An ultrastructural study of the submucous plexus of guinea pig intestine after unilateral vagotomy. J Anat.

[52] Lu J, Ng KC, Ling G, Wu J, Poon DJF, Kan EM, Tan MH, Wu YJ, Li P, Teo M, Yeh IB, Moochhala S, Yap E, Lee LKH, Sergio DMB, Chua F, Kumar D, Ling EA (2012). Effect of blast exposure on the brain structure and cognition in the Macaca fascicularis. J Neurotrauma.

[53] Paz Soldán MM, Pirko I (2012). Biogenesis and significance of central nervous system myelin. Semin Neurol.

[54] Deng YY, Lu J, Ling EA, Kaur C (2010). Microglia-derived macrophage colony stimulating factor promotes generation of proinflammatory cytokines by astrocytes in the periventricular white matter in the hypoxic neonatal brain. Brain Pathol.

[55] Kaur C, Sivakumar V, Ang LS, Sundaresan A (2006). Hypoxic damage to the periventricular white matter in neonatal brain: role of vascular endothelial growth factor, nitric oxide and excitotoxicity. J Neurochem.

[56] Nolan AM, Nolan YM, O’Keeffe GW (2011). IL-1β inhibits axonal growth of developing sympathetic neurons. Mol Cell Neurosci.

[57] Larsson K, Rydevik B, Olmarker K (2005). Disc related cytokines inhibit axonal outgrowth from dorsal root ganglion cells in vitro. Spine (Phila Pa 1976).

[58] Munoz L, Ralay Ranaivo H, Roy SM, Hu W, Craft JM, McNamara LK, Chico LW, Van Eldik LJ, Watterson DM (2007). A novel p38 alpha MAPK inhibitor suppresses brain proinflammatory cytokine up-regulation and attenuates synaptic dysfunction and behavioral deficits in an Alzheimer’s disease mouse model. J Neuroinflammation.

[59] Kellom M, Basselin M, Keleshian VL, Chen M, Rapoport SI, Rao JS (2012). Dose-dependent changes in neuroinflammatory and arachidonic acid cascade markers with synaptic marker loss in rat lipopolysaccharide infusion model of neuroinflammation. BMC Neurosci.

