# Supplementary figures and images for: Microglia-derived IL-1β contributes to axon development disorders and synaptic deficit through p38-MAPK signal pathway in septic neonatal rats
Source: J Neuroinflammation. 2017 Mar 14;14:52. doi: 10.1186/s12974-017-0805-x (PMC5348817; doi:10.1186/s12974-017-0805-x)

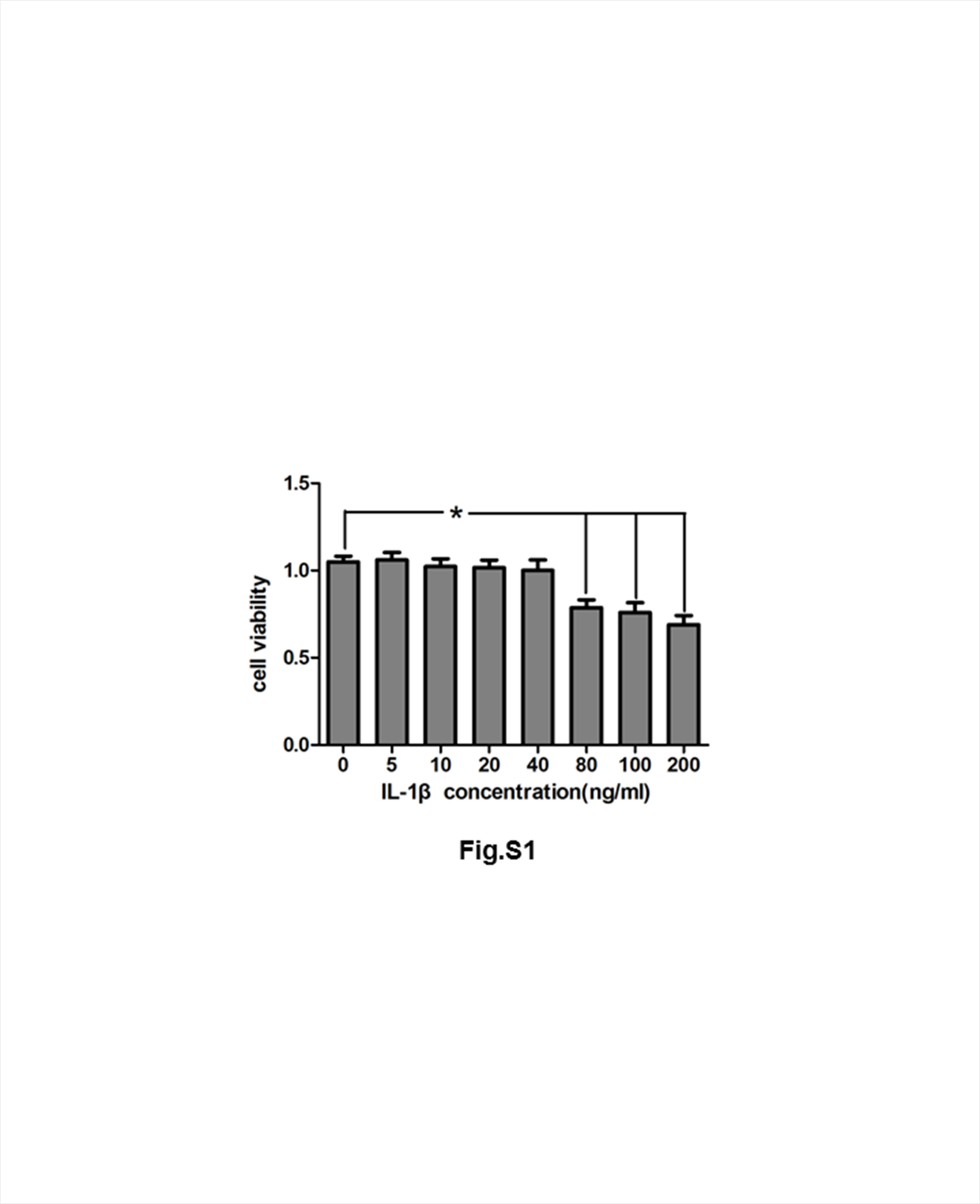

Supplement: Additional file 1: Figure S1. — The CCK-8 assay (cell counting kit 8) was performed to determine the IL-1β concentration. The viability of neuronal cells was significantly reduced when neurons were treated with IL-1β at a dose exceeding 40 ng/mL. (TIF 820 kb) [file 12974_2017_805_MOESM1_ESM.tif]

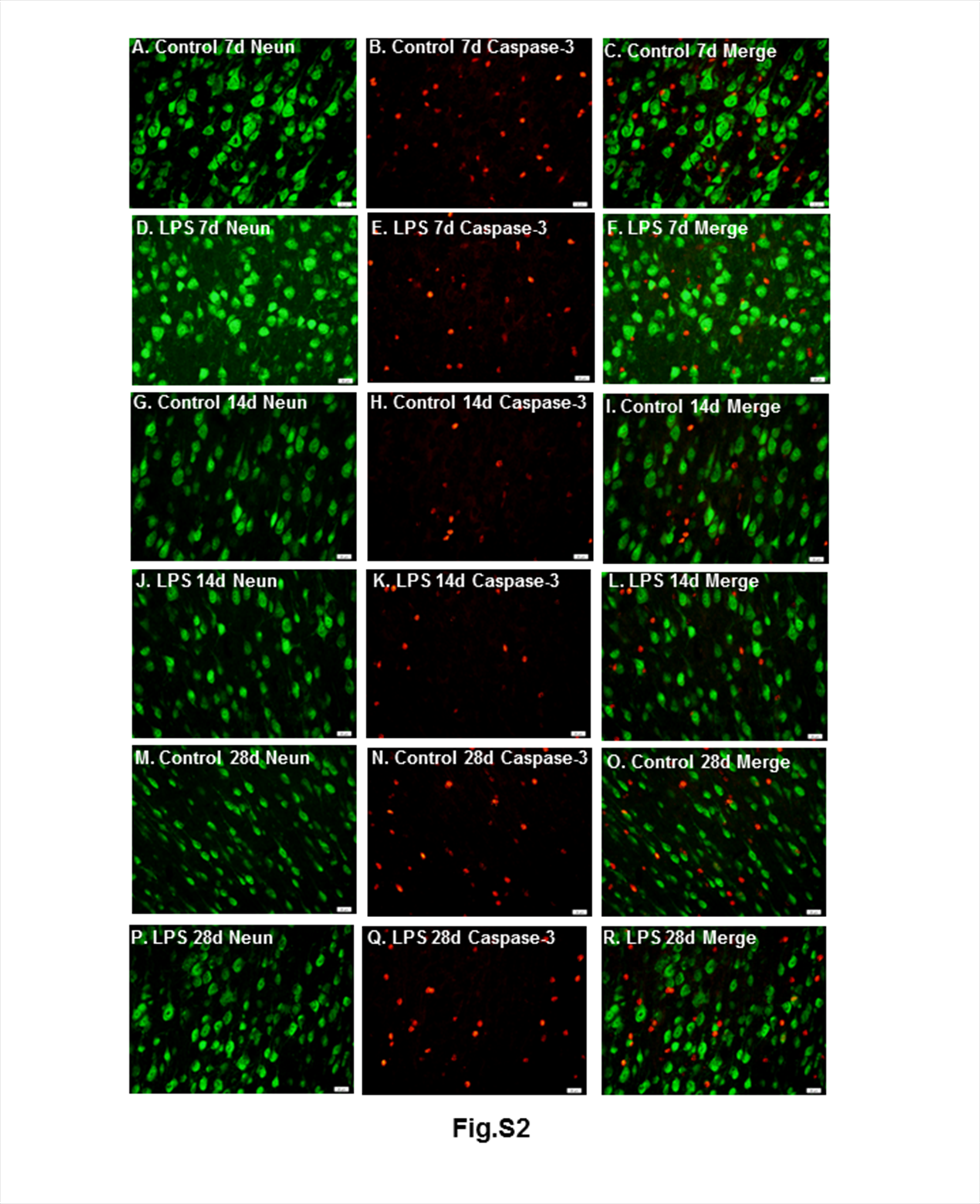

Supplement: Additional file 2: Figure S2. — Apoptosis of neurons in the cortex. The incidence of apoptotic neurons co-labeled by caspase-3 (red) and NeuN (green) did not change markedly at 7 days (A–F), 14 days (G–L), and 28 days (M–R) in the cerebral cortex after LPS injection (D–F, J–L, P–R) when compared with controls (A–C, G–I, M–O). Scale bars: A–R 20μm. (TIF 6590 kb) [file 12974_2017_805_MOESM2_ESM.tif]

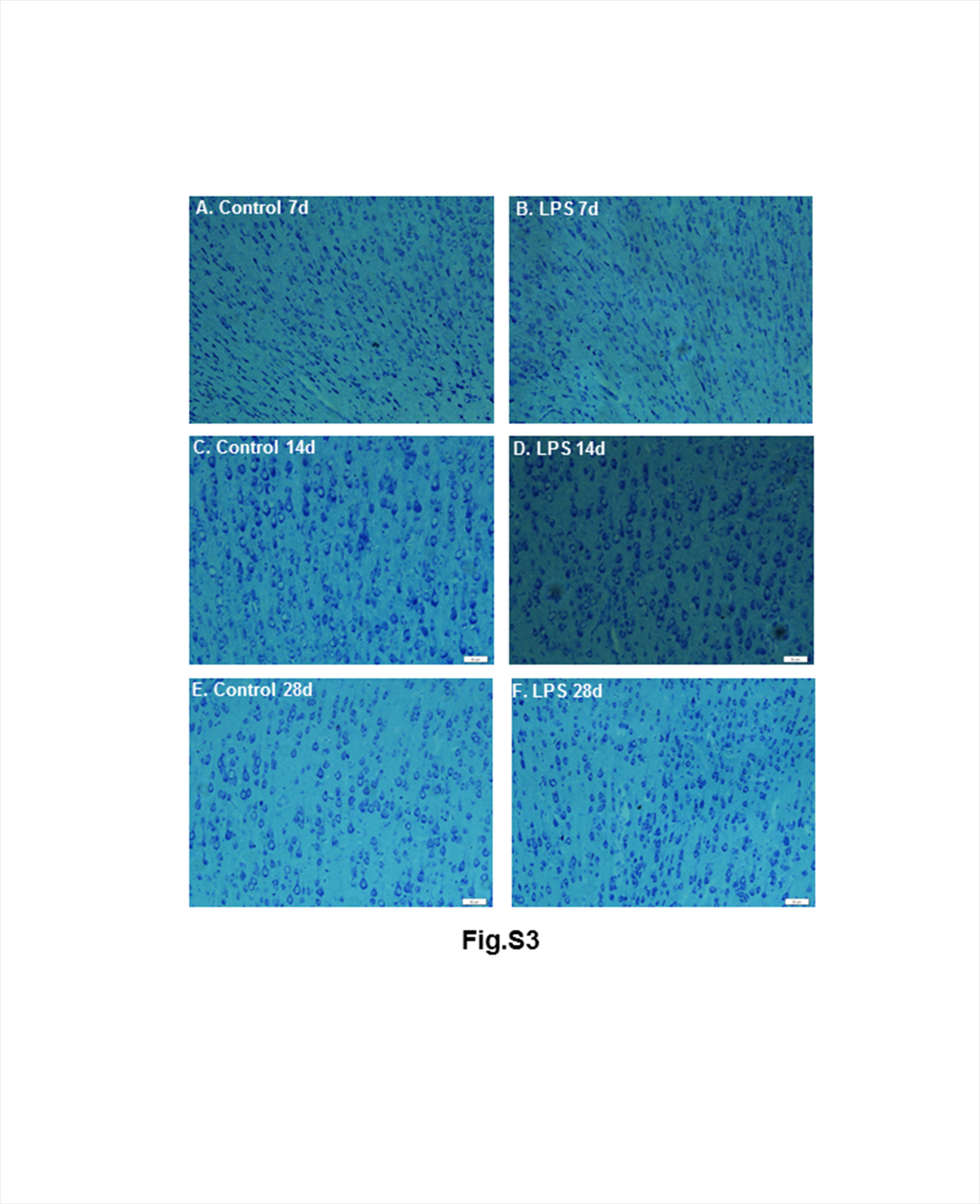

Supplement: Additional file 3: Figure S3. — Nissle staining shows the number of neurons in the cortex of postnatal rats at 7, 14, and 28 days after LPS injection (B, D, F) and their corresponding controls (A, C, E). (TIF 7048 kb) [file 12974_2017_805_MOESM3_ESM.tif]
